# Constrained Structure Minimizations on Hyperspheres for Minimum Energy Path Following

**DOI:** 10.1021/acs.jcim.4c02351

**Published:** 2025-04-01

**Authors:** Jorge Alberto Sanchez Alvarez, Luis López-Sosa, Andreas M. Köster, Patrizia Calaminici

**Affiliations:** Chemistry Department, CINVESTAV, Av. Instituto Politécnico Nacional 2508, Col. San Pedro Zacatenco, Del. Gustavo A. Madero, C.P. 07360 Mexico City, Mexico

## Abstract

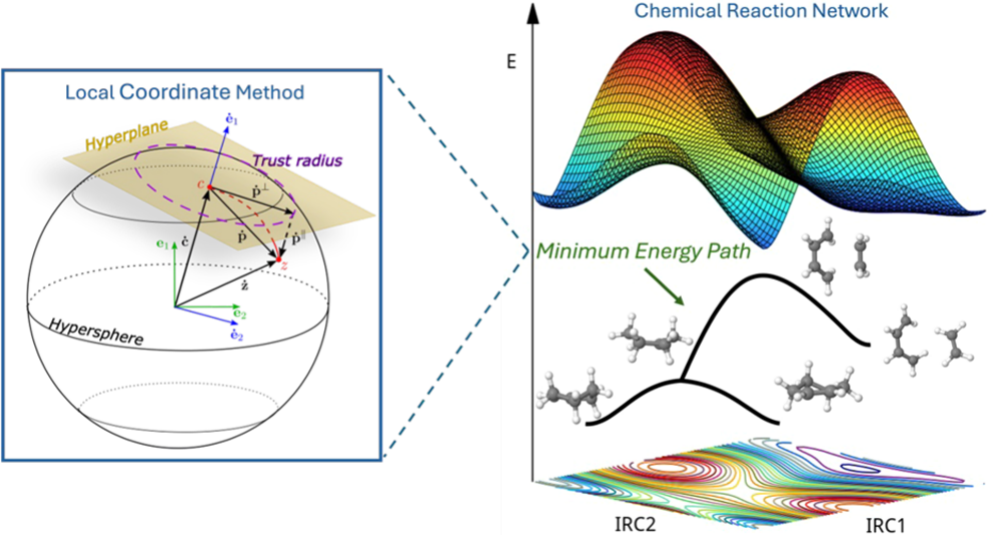

In this work, a reliable and robust trust region method for restricted minimizations on hyperspheres is developed. The working equations of this new methodology are presented, together with their validation. The performance and characteristics of this new algorithm are discussed by a constrained minimization on a sphere using a two-dimensional Quapp model surface. The obtained results show that the proposed method for minimizations on hyperspheres guarantees convergence to constrained minima. Its enhanced numerical stability permits tight convergence criteria for constrained minimizations. The application of the new restricted minimizer in the framework of the hierarchical transition state finder and for the calculation of intrinsic reaction coordinates for 38 chemical reactions demonstrates its robustness and efficiency.

## 1 Introduction

The theoretical description of stable structures and chemical reactions can become a complex task if the systems under study possess several stable isomers lying very close in energy. Typical examples are finite metal and transition metal clusters for which several stable isomers with similar energy may exist. These systems often present a strong dependency of their physical, chemical, and electronic properties on their size and geometric structure.^1,2^ For the automatized systematic structure optimization of (transition) metal clusters and compounds, machine learning algorithms^3^ and parallel tempering Born–Oppenheimer molecular dynamics simulations^4^ have shown promising results. In both cases, accurate local structure optimizations are needed to optimize stable minima that are often close in energy and have similar geometrical structures. Therefore, it is very important to be able to clearly distinguish one cluster structure from the others. Furthermore, it has been shown that the study of isomerization reactions of metallic clusters is important for the description of their properties and chemical reactivity because clusters of the same size but with different structures can have very different reactivity.^5,6^ Therefore, the study of these systems presents a theoretical challenge.

Theoretical studies of this type of finite systems require not only a detailed knowledge of their minimum energy structures but also of the corresponding isomerization network in order to understand their physicochemical properties.^7−11^ This implies a detailed study of the corresponding potential energy surface (PES). Today, first-principles density functional theory (DFT)^12,13^ methods are commonly employed due to their outstanding accuracy to performance ratio.^14^ In this work, we use a low-order scaling variant of Kohn–Sham DFT in the form of auxiliary density functional theory (ADFT)^15^ that is particularly well suited for large complex systems. It also provides numerically stable first- and second-order analytic derivatives^16^ that are mandatory for an efficient exploration of the PES. One way to characterize a PES is by the location of its relevant critical points (CPs). The CPs are characterized by the spectrum of the Hessian matrix, which consists of the second derivatives of the energy with respect to the nuclear positions. Reaction coordinates are described by minimum energy paths (MEP) on the PES that connects reactants and products through transition states (TS). More complicated reactions that include intermediates are broken down into sequences of such elementary reaction steps. This is the reason why the characterization of minimum energy structures (reactants and products) and first-order saddle points on the PES is of paramount importance to computational chemistry. Theoretical procedures for finding local minima on the PES are based on variants of the Newton and quasi-Newton methods that ensure convergence to a minimum rather than to other CPs of the PES.^17,18^ The main disadvantage of Newton method variants in first-principles electronic structure geometry optimizations is the explicit use of second-energy derivatives with respect to nuclear coordinates in each optimization step. Although the analytic Hessian matrix calculation in ADFT has been significantly improved in recent years by utilizing auxiliary density perturbation theory (ADPT),^16^ it still remains one order of magnitude slower than corresponding ADFT gradient calculations. Therefore, the calculation of the Hessian matrix in each optimization step increases substantially the computational time for first-principles electronic structure geometry optimizations. An alternative are quasi-Newton methods that avoid the analytical evaluation of the Hessian matrix in each optimization step. Instead, an update scheme is used, which modifies the Hessian matrix based on gradient and step size information. The efficiency and reliability of quasi-Newton structure optimizations depend on several factors, including the initial estimate of the Hessian matrix, the method of updating it, and finally, the control of the search direction and step size. To this end, restricted step algorithms (RSA) based on the Levenberg–Marquardt (LM) method^17−20^ combined with quasi-Newton Hessian updates have been successfully used to perform energy minimizations of a large variety of molecules including transition metal clusters.^21−25^ A particular example of this approach is the trust region (TR) method, which has been efficiently implemented in the quantum chemistry program deMon2k^26^ to perform molecular geometry optimizations.

On the other hand, due to the nature of a saddle point, the search for a TS is a more complicated task than the search of minimum structures on the PES. The two most common intuition free methods for the search of TSs on a PES are either single-ended (SE) approaches, which only need the information on one local minimum, i.e., the reactant or the product, to start the TS search, or double-ended (DE) approaches, which require the information on both structures, i.e., the reactant and the product of a reaction.^27^ The advantage of SE methods is that they work, even when the reactant or product of the reaction is unknown. Thus, they are of great value for discovering new reaction mechanisms and the local exploration of a complex PES with various local minima.

A pioneering SE method developed by Cerjan and Miller is the so-called eigenvector-following (EF) method.^28^ In this approach, a specific eigenvector (or mode) of the Hessian matrix is chosen to represent the reaction coordinate. The system is pushed along this mode to ascend in energy, while energy minimization is performed along all orthogonal directions. Usually, the eigenvector corresponding to the lowest eigenvalue, referred to as the minimum mode, is chosen as the reaction coordinate. As long as this assumption holds, the EF method works flawless. However, there are cases where the minimum mode does not accurately represent the reaction coordinate.^29^ In such instances, it may be necessary to select and follow a different eigenvector. Otherwise, the EF calculation may converge to an undesired TS. Therefore, the importance of the EF method has shifted over the years from a standalone SE approach to a critical ingredients of DE approaches such as the nudged elastic band,^30−33^ growing string,^34^ quadratic synchronous transit,^35,36^ and hierarchical transition state search^37^ methods, to name a few. The integration of the EF method into these approaches aims to refine the TS structures after initial interpolations. Even in modern SE approaches such as the scaled hypersphere search^38,39^ or a variant of the growing string^40^ method, it is necessary to employ a strategy to refine the optimized TS structure. Therefore, these methods only reach the quadratic region of a TS, i.e., the region where the Hessian matrix possesses one negative eigenvalue and all others are positive.

**Figure 1:**
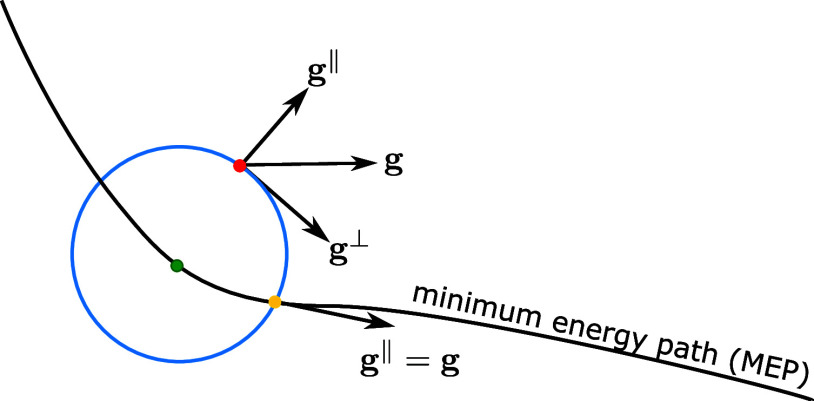
Schematic representation of the decomposition of the gradient, **g**, calculated at a point (red) on the surface of a hypersphere into its tangential, **g**^⊥^, and parallel, **g**^∥^, components. The blue circle represents a cut of a hypersphere and the curved black solid line represents the reaction path of a system. See text for further details.

To the best of our knowledge, Abashkin and Russo were the first authors who attempted a geometrically constructed SE TS search in first-principles electronic structure methods.^41^ Their approach was based on an older proposal by Dewar et al.,^42^ implemented in semiempirical electronic structure methods. The underlying idea is to perform restricted minimizations on a hypersphere by reducing the values of the tangential gradient in each minimization step. The end point of such a constrained minimization sequence is the point on the MEP that connects reactants and products. The particularity of this approach is that it is free of Hessian matrix calculations for the SE TS search. A schematic illustration is given in Figure 1. The blue circle in this figure represents a cut of the hypersphere that is constructed around the green point, which, in this case, is a point on the MEP, the solid black line. A gradient, **g**, calculated at the red point of the hypersphere, can be decomposed into its tangential, **g**^⊥^, and parallel components, **g**^∥^, respectively. At the crossing point of the hypersphere with the MEP, the yellow point in Figure 1, the tangential gradient vanishes, and therefore, **g**^∥^ = **g** holds. Later on, it was realized^38,39^ that this geometrical construction only holds for quadratic surfaces, which resulted in the scaled hypersphere search, a generalization of the Dewar method. In any case, this approach involves successive repetitions of restricted minimizations on consecutive hyperspheres, aimed to ascend the PES until a TS is located. The sequence of the minima points on the hyperspheres describes the reaction path of a system (curved solid line in Figure 1). Originally, the Dewar method was proposed using only gradient information. However, this methodology presents convergence problems or even complete failures. This occurs because the method relies solely on the gradient of the energy during the constrained minimization.

A variant of the Dewar method was used by Gonzalez and Schlegel (GS) for intrinsic reaction coordinate (IRC) calculations.^43^ The constrained minimization in the GS method differs from the hypersphere optimization technique proposed by Abashkin and Russo in that it employs the TR method with one undetermined Lagrange multiplier. However, different from unconstrained minimization, the Lagrange multiplier must now satisfy two constraints, namely, that the optimization step lies on the hypersurface and that the tangential part of the Hessian matrix is positive definite. Unfortunately, it is possible that one of the two conditions may not hold, which significantly increases the probability of method failure, as described in Section 2. Therefore, a more reliable algorithm for restricted minimization on hypersphers is needed. Such an algorithm is the prerequisite for the successful application of Dewar’s method and its generalizations to follow MEPs without Hessian matrix calculations. In this paper, the working equations of a new algorithm for restricted minimizations on hyperspheres are derived.

The article is organized as follows. After this introduction, Section 2 presents the theoretical formulation of the here proposed algorithm, followed by a comparison with the GS method for the restricted optimization on a circle on the Quapp model surface.^44^ Subsequently, in Section 3, the computational details of the application calculations are given. As applications for the newly developed restricted hypersphere minimization algorithm, we performed double-ended saddle interpolations within the hierarchical TS search and corresponding IRC calculations for a set of 38 representative reactions. These calculations are discussed and compared with available literature results in Section 4. Finally, in the last section, the conclusions are summarized.

## 2 Theory

In this section, we describe the newly developed restricted hypersphere LM minimization method. To put this description in context, we first briefly review the TR and GS methods in the next subsections.

### 2.1 Trust Region Method

The TR method first determines a maximum step length and then searches for a direction that best meets this step length constraint by solving the following minimization problem:
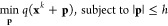
1The trust region is a hypersphere defined by |**p**| ≤ *h*, where the scalar *h* > 0 is the radius of the trust region. The model function, *q*(**x**^*k*^ + **p**), is defined by the Taylor series expansion up to second order around a point **x**^*k*^ on the PES:
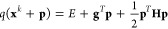
2In eq 2, *E* is the value of the objective function, in this case the energy of the system, **p** is the step vector, **g** is the gradient vector and **H** is the Hessian matrix or an approximation of it. All quantities are evaluated at point **x**^*k*^ on the PES. To solve eq 1, establishing **x**^*k*^ = 0, the following Lagrange function is introduced:
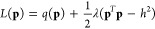
3

Here, λ is the undetermined Lagrange multiplier that must be calculated. When the Hessian matrix is positive definite and the step vector lies strictly inside the trust region, |**p**| < *h*, the optimal λ value for eq 3 is λ^(*)^ = 0, leading to the so-called Newton step. Otherwise, the value of λ must be determined in such a way that the augmented Hessian matrix becomes positive definite. From the stationary condition of the Lagrange function, the LM step vector is found as^22^
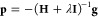
4

Note that this solution lies on *h*, i.e., |**p**| = *h*. If the solution of eq 1 does not decrease the energy function, then the trust region is too large and must be reduced. After the radius of the trust region is reduced, eq 1 is resolved again. This procedure is repeated until an acceptable decrease in the energy is achieved that satisfies a user-defined criteria.

### 2.2 Gonzalez–Schlegel Method

In the GS method for restricted minimization on a hypersphere, the same quadratic expansion of the energy as described by eq 1 is used. However, different from unconstrained minimization, the optimization step must now be restricted to stay on the hypersphere (blue circle in Figure 1) with radius . To this end, the constraint of the minimization problem is modified to
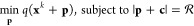
5

In eq 5, **c** is the vector with length  from the center of the hypersphere to the point **x**^*k*^ on its surface and **p** is the optimization step that is bound to the surface of the hypersphere. Again eq 5 is solved by introducing an undetermined Lagrange multiplier. From the stationary condition of the corresponding Lagrange function, the optimization step vector is calculated as
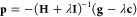
6The augmented inverse Hessian matrix that appears in eq 6 resembles the trust region step formula; see eq 4. However, unlike in the trust region formula, the hypersphere optimization step vector now also includes a modified gradient. Therefore, the undetermined Lagrange multiplier is chosen such that the optimization step always lies on the hypersphere. As a result, the positive definiteness of the augmented Hessian matrix is not always guaranteed.

### 2.3 Local Coordinate (LC) Method

**Figure 2:**
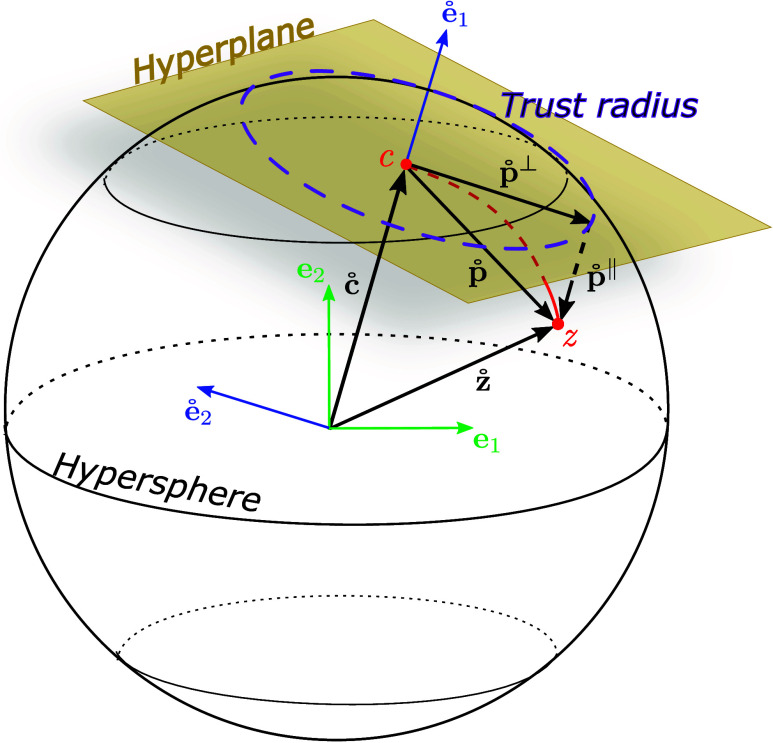
Schematic illustration of the transformation from global coordinates (green) to local coordinates (blue) in the LC method. The local direction, **e̊**_1_, is the normal vector to the tangential hyperplane drawn in yellow.

In order to address the two constraints, namely, the restriction of the optimization step to the hypersurface and the positive definitness of the tangential Hessian matrix for the minimum search, we propose the transformation of the minimization problem into a local coordinate system. The resulting local coordinate (LC) method is schematically depicted in Figure 2. In this approach, the global coordinate system (green unit vectors **e**_1_ and **e**_2_ in Figure 2) is transformed to a local one (blue unit vectors **e̊**_1_ and **e̊**_2_ in Figure 2) such that **e̊**_1_ becomes the normal vector of the tangential hyperplane in Figure 2. To do so, we apply for a nonlinear (linear) molecular system 3*N* – 7 (3*N* – 6) succesive orthogonal rotations to the vector **c** such that the resulting transformed vector **c̊** in the local coordinate system has only one nonvanishing component:
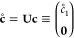
7with:
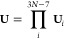
8

The algorithm for these rotations is outlined in the Supporting Information (SI). By construction, vector **c̊** is perpendicular to the hyperplane in point *c* given by the corresponding red dot in Figure 2. Due to our transformation, the first component of the vector **c̊** is equal to the radius of the hypersphere, and the other components of this vector are equal to zero. The transformation to the local coordinate system also projects the step vector, **p**, and the gradient vector, **g**, onto their parallel and tangential components. As already mentioned, the minimum on the hypersphere is characterized by its vanishing tangential gradient. Figure 2 shows that only through the tangential components a minimization on the hyperplane (yellow plane in Figure 2) tangential to the hypersphere can be performed. Therefore, only the tangential components are considered in the quadratic expansion, and consequently, the equation of the model function can be written as
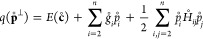
9

Note the summation starts with *i*, *j* = 2, which eliminates the parallel components from the minimization problem. The upper limit is *n* = 3*N* – 6 (3*N* – 5) for nonlinear (linear) molecules. The transformed quantities in the local coordinate system are denoted by circles and are calculated as
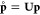
10a
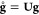
10b
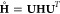
10c

As Figure 2 shows, minimization is performed in the hyperplane tangential to point *c*. Therefore, the minimization problem can be formulated as
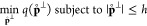
11

Employing the TR method, we solve this problem by introducing a Lagrange function and establishing a stationary condition for this function. Therefore, the LM step for constrained minimization in the hyperplane is calculated as
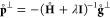
12

Since this step vector is calculated by the TR method, it ensures that a local minimum is always found in the hyperplane. The value of λ must be determined in such a way that the augmented Hessian matrix, **H̊** + λ**I**, is positive definite. This enforces the search for a minimum in the hyperplane. To connect the LM step in the hyperplane with the hypersphere, a correction step is needed. This correction step is given by the first component of the step vector in local coordinates as
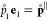
13

In Figure 2, the correction step ends at the red point *z* on the hypersphere. During the minimization the correction step decreases. In particular, the correction step vector tends to zero when the algorithm converges.

**Figure 3:**
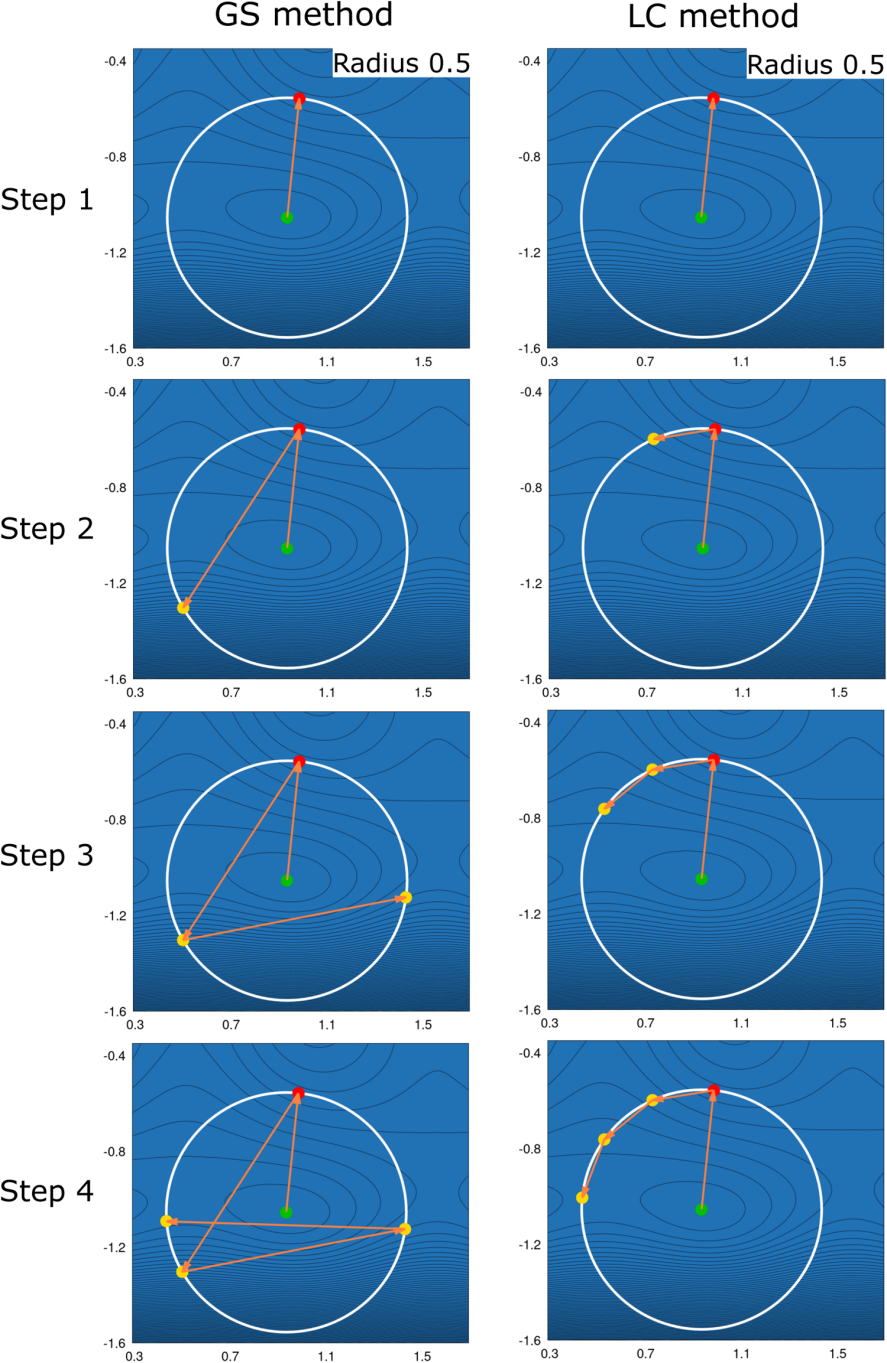
Sphere minimization steps of the GS and LC methods on the two-dimentional Quapp model surface. The green point represents a minimum on this surface. We use it as center for the white sphere. The red point represents the starting point on the sphere and the yellow points represent the performed restricted minimization steps.

The performance of the newly developed LC algorithm for restricted minimizations on a hypersphere was tested on the two-dimensional Quapp^44^ model surface. The results obtained with the LC method are compared with those from the GS method. To this end, we depict in Figure 3 successive steps with the GS (left) and LC (right) methods on the contour plots of the Quapp model surface. The local minimum whose coordinates are (0.94, −1.05), indicated by the green dots in Figure 3, was taken as the center of a sphere with a radius of 0.5 (white circles in Figure 3). The initial point for the start of the restricted minimization is the red point on the sphere. The minimization steps are illustrated with yellow points on the sphere. The first four steps are depicted in Figure 3. The optimization variables for nine optimizations are listed in Tables 1 and 2 for the GS and LC methods, respectively. Note that the gradients and Hessian matrices of the GS method refer to the global coordinate system, whereas for the LC method, they refer to the local coordinate system. The point obtained after applying the minimization step is denoted as **x**^*k*+1^ in these tables. In the last columns of these two tables, the norm of the tangential gradient is listed. For successful minimization, this norm must vanish. As can be seen from Table 1, the tangential gradient norm oscillates in the GS method hampering its convergence. This is also seen from the left panel of Figure 3 by the oscillation of the yellow points. The reason for this behavior lies in the mismatch between the number of constraints (2) and the undefined Lagrange multipliers (1).

**Table 1:** Optimization Variables of the GS Method for the Quapp Model Surface Example^a^

Iter.	point, **x**^(*k*)^	gradient, **g**	Hessian matrix, **H**	step, **p**	point, **x**^(*k*+1)^	|**g**^⊥^|^(*k*+1)^
1						18 253.10
2						4750.77
3						3032.05
4						351.65
5						5271.42
6						1244.48
7						4070.65
8						645.69
9						4596.63

a See text and Figure 3 for further details.

**Table 2:** Optimization Variables of the LC Method for the Quapp Model Surface Example^a^

Iter.	point, **x**^(*k*)^	local gradient, **g̊**	local Hessian matrix, **H̊**	local step, **p̊**	point, **x**^(*k*+1)^	|**g**^⊥^|^(*k*+1)^
1						2515.29
2						1405.02
3						53.77
4						1.52
5						6.40 × 10^–2^
6						2.65 × 10^–3^
7						1.10 × 10^–4^
8						4.58 × 10^–6^
9						1.90 × 10^–7^

a See text and Figure 3 for further details.

With the LC method, this mismatch is resolved. As Table 2 shows, the tangential gradient norm reduces in each optimization step and converges smoothly in 9 steps below 10^–6^. This smooth convergence can also be seen in the right panel of Figure 3 for the first 4 optimization steps. Because the step vector is calculated according to eq 9, only the second component of the local gradient vector, *g̊*_2_, and the *h̊*_22_ element of the local Hessian matrix are of relevance. Note that *h̊*_22_ is negative in the first step, which indicates a nearby maximum (higher order critical point) on the sphere. Due to the use of the TR method, the optimization step moves away from this critical point toward a minimum. Inspecting the local gradient in Table 2 shows a diminishing of the second component analogous to the tangential gradient norm. This is expected because only this component contributes in this example to the tangential gradient norm. The first gradient component represents the parallel gradient **g**^∥^, which remains at a large value (−437.31) at convergence. This is the gradient component in the direction of the MEP as can be anticipated from the bottom right graph in Figure 3. Finally, the local step vector in Table 2 increases and decreases during the constrained minimization with the LC method, as expected for a TR approach. As already outlined, the first component of the step vector represents the correction step. Initially, this step can be large. However, it decreases with the convergence of the algorithm and becomes zero in the last 2 steps. As this example shows, the LC method transfers the numerical robustness of the TR approach to the constrained minimization on hyperspheres. Although we used here calculated Hessian matrices, we will see in the Section 4 that this numerical robustness holds for quasi-Newton variants of the LC method, too.

## 3 Computational Details

**Figure 4:**
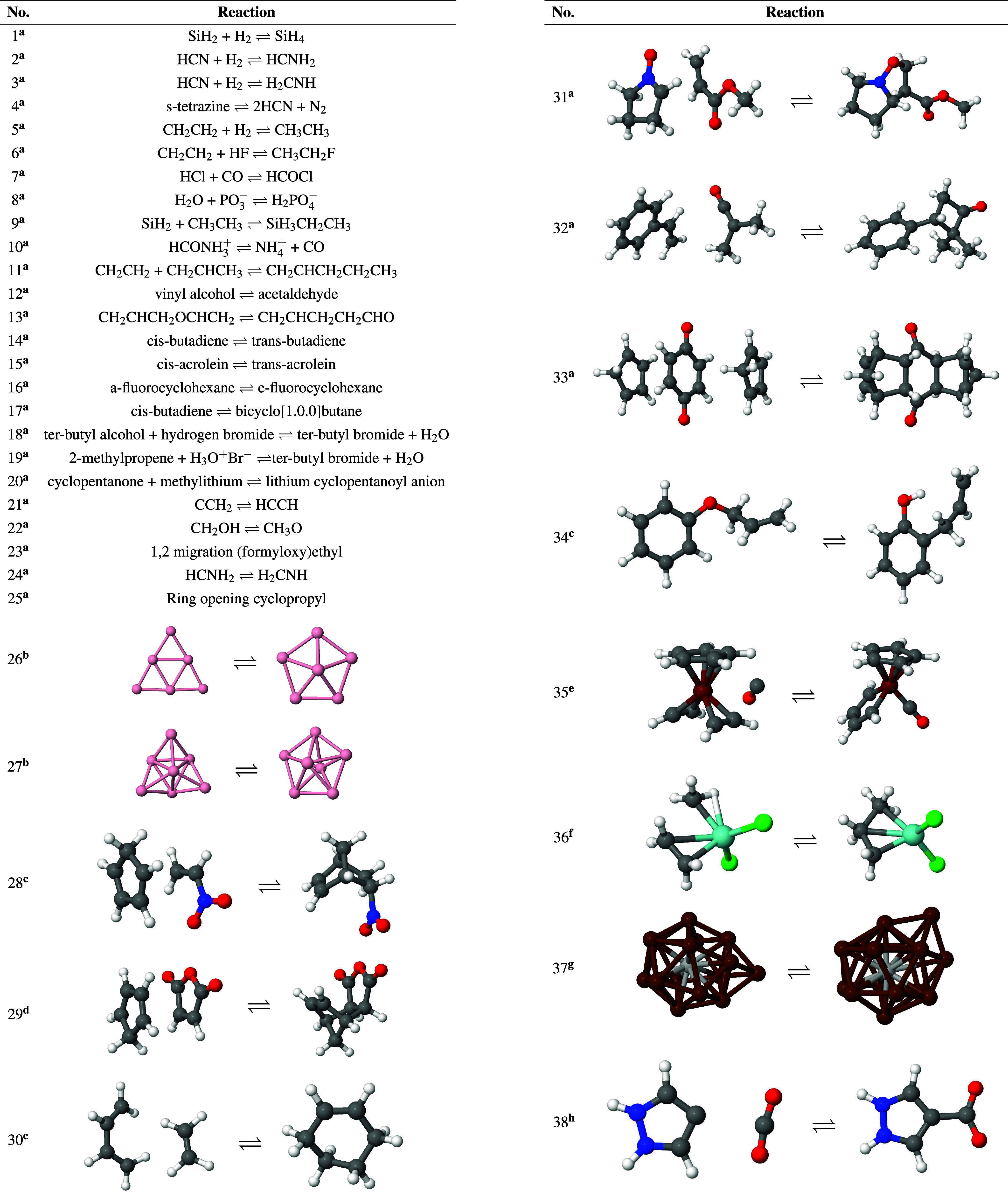
^a^From ref (37); ^b^From ref (8); ^c^From ref (58); ^d^From ref (59); ^e^From ref (60); ^f^From ref (61); ^g^From ref (51); ^h^From ref (62). Test set reactions for the application of the LC method in the saddle interpolations and IRC calculations. The reaction numbering is given, too. As atom colors we use white for hydrogen, gray for carbon, blue for nitrogen, red for oxygen, pink for sodium, maroon for cobalt, turquoise for zirconium, green for chlorine, brown for copper, and silver for vanadium.

All molecular calculations were carried out employing auxiliary density functional theory (ADFT) as implemented in the deMon2k program.^45^ The Coulomb energy was calculated using the variational fitting procedure proposed by Dunlap et al.^46^ The Perdew–Burke–Ernzerhof (PBE)^47^ exchange–correlation functional was used for the study of reactions 1–37 depicted in Figure 4. The M06^48^ hybrid exchange–correlation functional was employed for reaction 38 of Figure 4 because with PBE the reactant optimized barrierless into the product structure. The Zr atom in reaction 36 was treated with a Stuttgart–Dresden^49^ effective core potential (ECP) and a corresponding valence basis set, while the remaining atoms were described with the all-electron double-ζ valence plus polarization basis set (DZVP-GGA).^50^ The exchange–correlation energy and potential contributions were numerically integrated on a fixed fine grid. The GEN-A2* auxiliary function set was employed in all calculations.^50^ The local minima structures were fully optimized by using a quasi-Newton TR method in redundant internal coordinates. For these optimizations, the convergence criteria of the root-mean-square and largest gradient component were set to 1.0 × 10^–5^ and 1.5 × 10^–5^ au, respectively. The obtained local minima structures were characterized by a frequency analysis. In the saddle interpolation, the reaction coordinate distance between the reactant and product was reduced by 5 and 10% in each step, moving the lower energy structure toward the higher energy end point. This procedure was repeated until one of the following conditions was fulfilled. First, the reaction coordinate distance between the two structures became less than 0.1 au. Second, the cosine of the angle between the gradient vectors of the two end points reached a value between 0.7 and 1.0, and the energy of the constrained optimized structure was lower than the energy of either of the two end points. Satisfying the second condition indicates that the TS was overstepped. If the saddle interpolation method converged by overstepping, then the second last structure was taken as the starting point for the TS optimization. The constrained hypersphere minimizations with the LC method were performed in normal coordinate space.^51,52^ As the start Hessian, the identity matrix was used for all saddle calculations. During the constrained hypersphere minimization the Hessian matrix was updated with the BFGS formula.^53−56^ The default convergence criteria of the deMon2k program (3 × 10^–4^ au for rms gradient) were used in the constrained hypersphere minimizations for the saddle interpolations. Following the hierarchical TS search protocol,^37^ the approximated TS structure from the converged saddle interpolation was further optimized with the uphill trust region method in delocalized internal coordinates.^37^ For this local optimization of the TS, the start Hessian was calculated and updated with the POWELL method.^57^ The optimized TS was characterized by a frequency analysis. Beginning from the TS as the center of a hypersphere, the IRC was calculated by employing the LC method for the constrained hypersphere minimizations. Subsequently, the LC hypersphere minimization method was employed to descend on the PES and find local minimum structures. The maximum allowed IRC step size was set to 0.1 au. The convergence criteria of the root-mean-square and largest component gradient were set to 1.0 × 10^–5^ and 1.5 × 10^–5^ au, respectively. These thresholds were applied to both the constrained hypersphere minimization process and to the end points of the IRC calculation. The IRC calculations were initialized by analytical Hessian matrix calculations.

## 4 Applications

This section presents and discusses the results of the double-ended saddle interpolation method and IRC calculations employing the LC method for the underlying constrained hypersphere minimizations. To this end, all studied reactions are listed in Figure 4 together with their numbers used throughout the discussion. The reactions depicted in Figure 4 include dissociation, addition, rearrangement, internal rotation, and ring-opening reactions. The initial molecular coordinates were taken from refs (8,37,51,58−62).

### 4.1 Double-Ended Saddle Method for Transition State Search

Table 3 shows the number of saddle steps needed to approximate the TS structure for the reactions in the test set depicted in Figure 4. The table compares the number of saddle steps required when the reaction coordinate distance is reduced by 5 and 10%. These results are compared with the original double-ended saddle implementation in deMon2k, which uses the Gonzalez–Schlegel method for constrained hypersphere minimizations, as described by del Campo et al.^37^ Note that the saddle interpolation with the LC method using the same 5% reduction, as in ref (37),^37^ converges for all reactions faster than its Gonzalez–Schlegel analog. In total (sum entry of Table 3), a reduction of the saddle step by more than 25% is observed. An even larger reduction of saddle steps by almost a factor of 2 is found when the reduction of the reaction coordinate distance is enlarged to 10%. With the LC approach, such large reduction values become possible due to the enhanced numerical stability of the underlying constrained minimizations on hyperspheres. This highlights the efficiency of the hierarchical transition state finder in combination with the LC method, offering a significant improvement in performance over previous implementations. Furthermore, the LC method requires only energy and gradient calculations, which keeps the computational demand with ADFT more than manageable. This efficiency is achieved by starting all saddle calculations with a unit Hessian matrix, which is then updated using the BFGS procedure. Consequently, the saddle interpolation combined with the LC method did not require any Hessian calculation to efficiently follow the reaction path. As already mentioned in Section 3, the M06 functional was employed for reaction 38, as it is capable of describing the nitrogen heterocyclic carbene with the CO_2_ molecule as a reactant. Therefore, we employed the M06 functional for the saddle interpolation as well as for the local TS optimization and IRC calculations for this reaction.

**Table 3:** Number of Saddle Steps for the Test Set Reactions Shown in Figure 4, Calculated Using 5 and 10% Reduction Values for the Reaction Coordinate Distance^a^

	saddle steps				saddle steps		
No.	5%	10%	ref (37)	No.	5%	10%	ref (37)
1	54^b^	34^b^	86	20	70^b^	61^b^	
2	58^b^	42^b^	59	21	50^b^	40^b^	59
3	59^b^	47^b^	60	22	34^b^	28^b^	36
4	66^b^	47^b^	96	23	37^b^	32^b^	69
5	51^c^	38^c^	93	24	38^b^	29^b^	71
6	55^b^	42^b^	63	25	55^b^	42^b^	59
7	51^b^	40^b^	79	26	36^b^	30^b^	
8	61^b^	45^b^	69	27	42^b^	26^b^	
9	45^b^	37^b^	81	28	54^b^	35^b^	
10	56^b^	43^b^	78	29	47^b^	33^b^	
11	67^b^	29^c^	93	30	75^b^	48^b^	95
12	50^b^	40^b^	60	31	66^c^	61^c^	
13	84^c^	32^c^	104	32	68^c^	44^c^	
14	48^b^	33^b^	91	33	79^c^	36^c^	
15	48^b^	38^b^	66	34	73^c^	48^c^	
16	39^c^	22^c^	59	35	89^b^	48^b^	
17	36^b^	19^b^	38	36	38^b^	50^b^	
18	68^b^	35^b^		37	48^b^	29^b^	
19	72^b^	49^b^		38	48^b^	29^b^	
				sum	1217	847	1664

a Values are compared with those from ref (37), where a 5% reduction was employed, too. Only reactions directly comparable to those in ref (37) are included in the summation of saddle step values.

b Saddle convergence by distance criterium.

c Saddle convergence by overstepping.

Table 4 lists the number of EF optimization steps for the TS localization. According to the hierarchical TS search protocol, this optimization starts with the converged structure from the saddle interpolation for which the Hessian matrix is calculated. The listed optimization steps refer to saddle interpolation starting structures from 5% reduction. The corresponding numbers from the 10% reduction are similar. The EF follows the eigenvector corresponding to the negative eigenvalue of this Hessian employing POWELL updates^57^ in the following optimization steps. The third and fourth columns of Table 4 report the calculated activation energy barriers (Δ*E*_a_) and reaction energies (Δ*E*_rxn_), respectively. The Δ*E*_a_ was calculated as the energy difference between the reactants and TS structures according to the chemical reaction equations in Figure 4. Similar Δ*E*_rxn_ was determined by the energy difference between the product and reactant structures of these chemical reactions. The values in parentheses refer to zero-point corrected energies. To guarantee tight convergence of the TS structures, the convergence threshold for the root-mean-square gradient was set to 10^–5^ au in these optimizations. The Cartesian coordinates of the optimized TS structures are listed in the SI. Notably, TS structures were found for all reactions with very moderate numbers of optimizations steps. This underlies the quality of the starting structures from the saddle interpolations. Table 4 shows that reaction 36 requires the largest number of optimization steps to refine the TS structure due to the reorientation of the ethylene structure during local TS optimization. We also point out that reactions 26 and 27 are isomerizations of sodium clusters (see Figure 4) with small activation barriers and nonintuitive TS structures. The finding of such transition states is notoriously problematic but straightforward, with the hierarchical transition state finder employing the LC approach for the constrained minimizations on hyperspheres in the saddle interpolation.

**Table 4:** Number of Transition State Optimization Steps Together with the Corresponding Activation (Δ*E*_a_) and Reaction (Δ*E*_rxn_) Energies for the Test Set Reactions of Figure 4^a^

No.	optimization steps	Δ*E*_a_		Δ*E*_rxn_		No.	optimization steps	Δ*E*_a_		Δ*E*_rxn_	
1	2	3.29	(1.84)	–47.61	(−46.48)	20	23	0.43	(0.87)	–26.28	(−22.71)
2	3	65.87	(59.59)	–12.77	(−19.80)	21	4	37.55	(33.91)	–5.27	(−3.13)
3	3	73.14	(73.77)	–22.97	(−15.37)	22	2	18.32	(15.56)	–35.18	(−35.62)
4	4	45.29	(40.65)	–13.34	(−20.31)	23	5	111.24	(102.64)	–14.17	(−13.98)
5	18	71.66	(73.04)	–42.14	(−34.43)	24	18	25.57	(22.56)	–32.75	(−32.68)
6	2	38.47	(36.78)	–16.49	(−13.48)	25	7	24.86	(23.04)	–25.80	(−26.12)
7	3	27.80	(28.05)	–9.22	(−6.02)	26	14	2.33	(2.27)	–0.78	(−0.76)
8	9	14.48	(15.17)	–18.19	(−17.75)	27	4	0.61	(0.55)	–2.76	(−2.76)
9	5	15.54	(14.16)	–45.93	(−45.55)	28	3	7.38	(8.63)	–28.05	(−23.28)
10	12	29.37	(26.04)	–11.68	(−12.62)	29	7	8.23	(9.17)	–24.51	(−20.30)
11	4	23.63	(23.26)	–25.85	(−22.40)	30	28	12.73	(14.24)	–49.90	(−44.13)
12	3	51.13	(47.18)	–10.12	(−10.81)	31	29	8.94	(9.82)	–16.40	(−13.01)
13	9	52.26	(48.62)	–18.88	(−19.25)	32	24	14.52	(14.96)	–25.68	(−22.73)
14	2	3.94	(3.51)	–3.71	(−3.65)	33	4	9.12	(10.06)	–35.21	(−27.18)
15	19	6.87	(6.18)	–2.24	(−2.24)	34	19	55.06	(50.92)	–13.51	(−13.39)
16	28	26.08	(26.27)	–73.90	(−69.50)	35	24	21.01	(20.13)	–56.83	(−57.71)
17	15	41.70	(40.26)	–17.80	(−18.67)	36	55	0.27	(1.40)	–12.83	(−9.88)
18	16	37.50	(36.56)	–2.21	(−1.45)	37	14	5.14	(5.20)	–2.20	(−1.94)
19	5	15.24	(14.23)	–14.00	(−11.12)	38	7	1.04	(0.98)	–10.91	(−9.34)

a Values in parentheses are zero-point corrected. All energy values are in kcal/mol. See text for further details.

### 4.2 Intrinsic Reaction Coordinate Calculations

**Figure 5:**
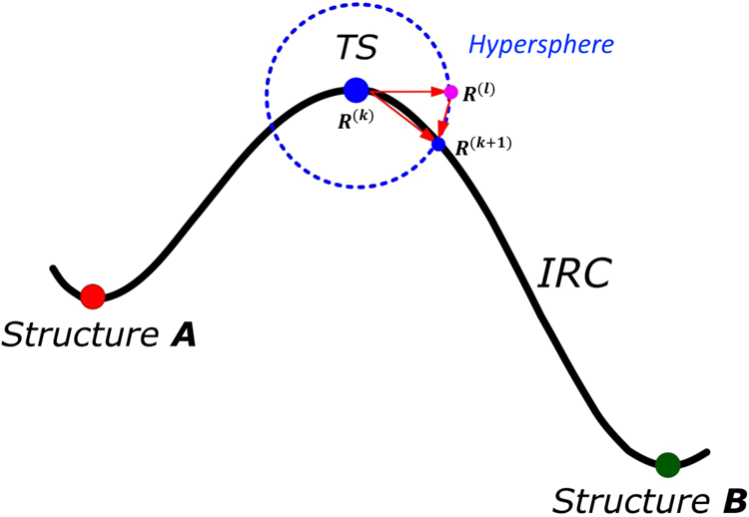
Schematic representation of the LC method for IRC calculations.

To fully identify the mechanism of a chemical reaction, the TS optimization and characterization must be followed by IRC calculations to verify that the obtained TS structure indeed connects the original reactant and product valleys of the PES. In recent years, various methods for obtaining IRC paths, such as the predictor–corrector and Hessian-based predictor–corrector reaction path following integrator for evaluating scalar curvature profiles, have been explored.^63^ It has been demonstrated that both predictor–corrector schemes result in pathways exhibiting scalar curvature profiles with significant quantitative and qualitative errors.^64^ Therefore, we use here the LC method to calculate the IRC as schematically depicted in Figure 5. The dashed blue line represents the hypersphere surface around the TS structure located at **R**^(*k*)^. The crossing point between the TS eigenvector and the hypersphere, **R**^(*l*)^, is used as a starting point for the LC optimization on the hypersphere. The new coordinates after constrained minimization are given by **R**^(*k*+1)^. The optimization step is calculated using eqs 12 and 13. Once the LC method converges to a minimum on the hypersphere, the optimized molecular structure lies on the hypersphere and on the IRC path. Therefore, **R**^(*k*)^ and **R**^(*k*+1)^ represent the optimized IRC step. As a result, all IRC plots are smooth and continuous around the TS structure. The IRC plots of all reactions illustrated in Figure 4 are given in the SI. In all IRC profiles, the uncorrected relative energy (in kcal/mol) is plotted against the mass-weighted IRC coordinate (in amu^1/2^ Å). We now discuss some IRC calculations in more details.

**Figure 6:**
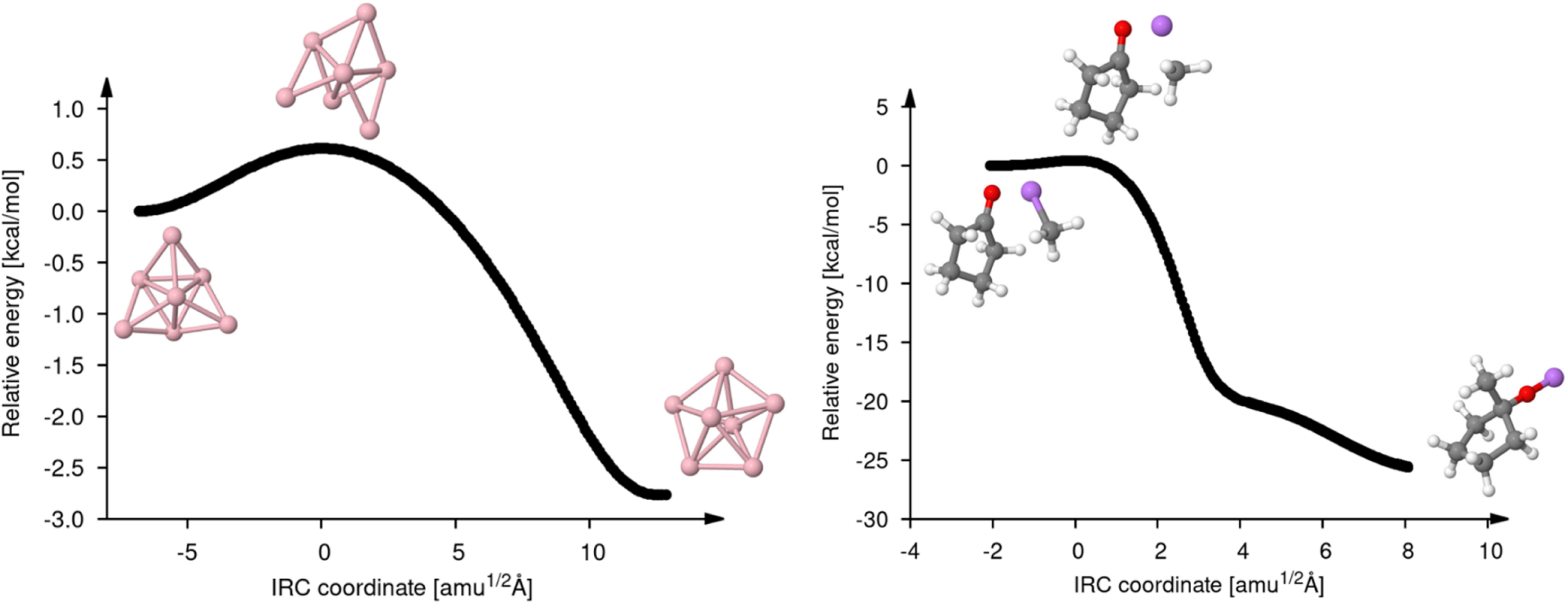
IRC plots for reaction 27 (left) and 20 (right) from Figure 4. The uncorrected relative energy [kcal/mol] is plotted against the mass-weighted IRC coordinate [amu^1/2^ Å]. The TS is taken as origin for the IRC coordinate. Note the difference in the energy scales for the two reactions.

Figure 6 presents the IRC plots for reactions 27 and 20. We selected these two reactions because of their low activation barriers. This makes TS finding challenging and also demands tight hypersphere minimizations during the IRC calculations. The left panel of Figure 6 depicts the IRC path for the Na_7_ cluster rearrangement, with which we will start the discussion. As this figure shows, the Na_7_ rearrangement occurs in a small energy window well below 5 kcal/mol. The activation energy is only 0.61 kcal/mol (see Table 4) and the corresponding reaction energy is −2.76 kcal/mol. Despite these small energy differences, the hierarchical TS finder with the LC method in the saddle interpolations found the TS and a smooth IRC path was obtained again employing the LC method. An even more challenging TS search appears in reaction 20 (right panel of Figure 6), in which a cyclopentanone reacts with a methylithium, due to the large unbalance between a small activation barrier of 0.43 kcal/mol and a much larger reaction energy of −26.28 kcal/mol. It is not uncommon that in such cases, TS finders overstep the TS structure. Again, this is not the case with the hierarchical TS finder employing the LC method in the saddle interpolation. Also, the IRC calculation with the LC method for this reaction provides a smooth IRC path as the right panel of Figure 6 shows.

**Figure 7:**
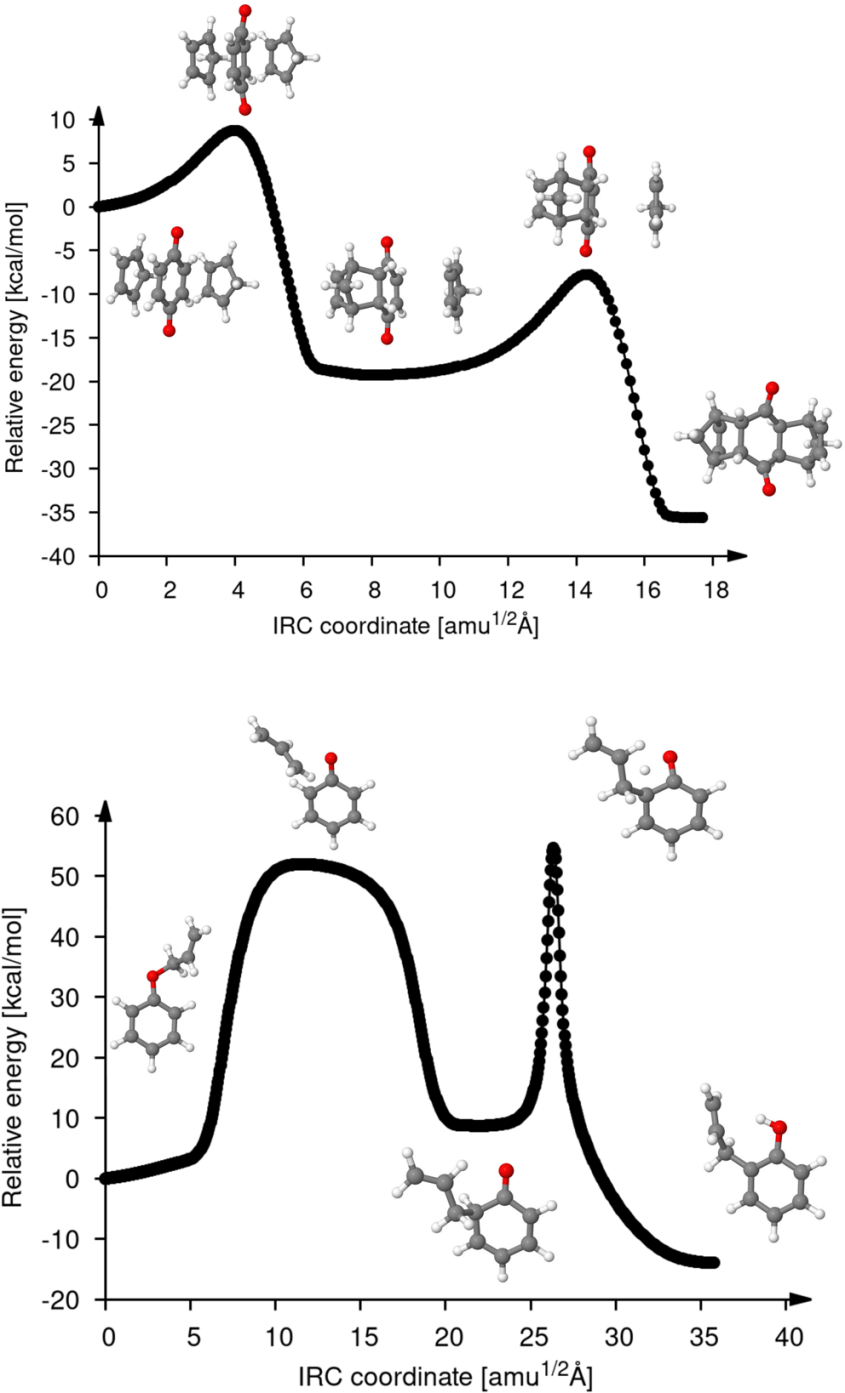
IRC plots for reactions 33 (top) and 34 (bottom) from Figure 4. The uncorrected relative energy [kcal/mol] is plotted against the mass-weighted IRC coordinate [amu^1/2^ Å]. The reactant is taken as origen for the IRC coordinate. Note the difference in the energy scales for the two reactions.

Figure 7 shows the IRC plots for reactions 33 and 34. We selected these two reactions, because they proceed through intermediates. The top panel of Figure 7 shows the Diels–Alder cycloaddition of two cyclopentadienes with 1,4-benzoquinone. As expected, these cycloadditions proceed stepwise, one after the other, which results in the shown intermediate. The relative barriers and reaction energies for both additions are very similar. The overall activation energy (for the first cycloaddtion) is 9.12 kcal/mol, and the corresponding reaction energy is −35.21 kcal/mol (see Table 4). The bottom panel of Figure 7 shows the Claisen rearrangement of allyl phenyl ether. The allyl phenyl ether rearrangement proceeds through a pericyclic mechanism in which a reorganization of the bonding electrons occurs through a cyclic TS (first TS structure in the bottom panel of Figure 7). Subsequently, 6-allyl-2,4-cyclohexadienone is formed as an intermediate. In the following reaction step, this intermediate undergoes keto–enol tautomerism to isomerize to *o*-allylphenol. This isomerization passes through the highest energy barrier of the entire reaction given by the second sharp peak in the bottom panel of Figure 7. The sharpness of this reaction barrier suggests significant proton tunneling in the transfer step. Thus, the calculation of the full IRC path for the allyl phenyl ether rearrangement provides insight into the proton transfer mechanism of this second part of the reaction, too. The formal Δ*E*_a_ of reaction 34 is 55.06 kcal/mol and the Δ*E*_rxn_ is −13.51 kcal/mol. These examples show that the hierarchical transition state finder in combination with IRC calculations, both employing the LC method, can correctly describe reactions with intermediate structures.

To determine the full IRC pathway for these two reactions with intermediates, we proceeded as follows: First, the saddle method was employed for each reaction using as input structures the reactants and products illustrated in Figure 4. The structures obtained from each saddle interpolation were used as the starting geometries for subsequent local TS optimization by using the EF method, followed by characterization via frequency analysis. In both cases, the TS with the higher energy was obtained. For reaction 33, the first found TS structure possesses an activation energy of 9.12 kcal/mol. This structure corresponds to the first TS appearing in the top panel of Figure 7. Similarly, for reaction 34, the first found TS structure possesses an activation energy of 55.06 kcal/mol, corresponding to the second TS shown in the bottom panel of Figure 7. Starting from these TS structures, IRC calculations were performed. As can been seen from Figure 7 for reaction 33, the resulting IRC pathway connects the reactant structure to an intermediate, whereas for reaction 34, the resulting IRC pathway connects the product structure to an intermediate. The intermediate structures identified in both cases were then optimized and characterized through frequency analysis, confirming them as minima. Subsequently, a second saddle interpolation was performed for each reaction. For reaction 33, the saddle interpolation used the product and intermediate structures as input structures, while for reaction 34, the reactant and intermediate structures were used. The TS starting structures obtained from each saddle interpolation were then further optimized and characterized through frequency analysis, finding the second TS for both reactions. Finally, using these second TS structures, a second IRC calculation was performed to establish the connection between the intermediate and the end structures in each reaction. In this way, we were able to obtain the complete reaction pathway for both reactions as ilustrated in Figure 7.

**Figure 8:**
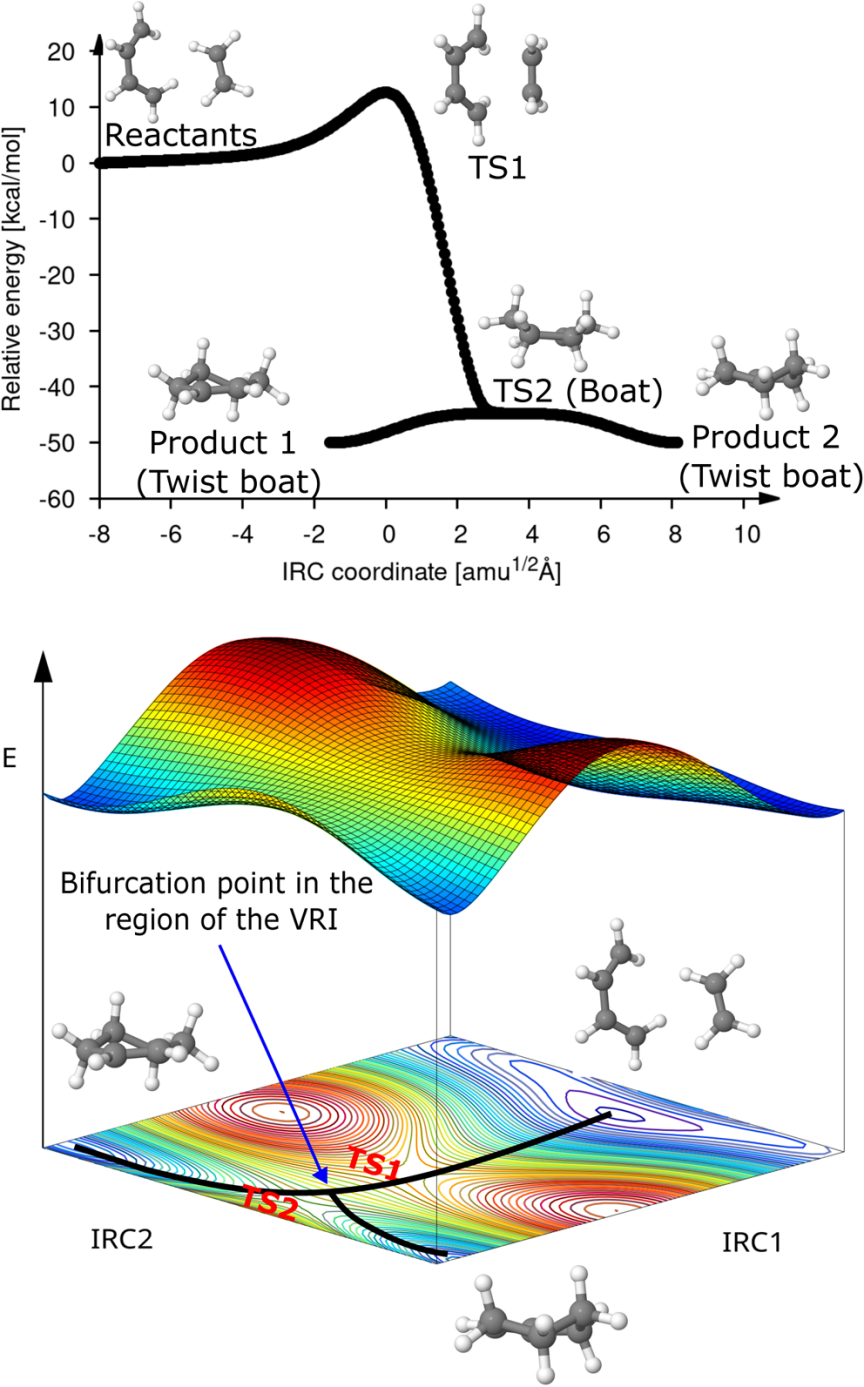
Top: IRC plot of reaction 30 with two consecutive TS structures (TS1 and TS2) linking the reactants with two products. Bottom: Model PES featuring a valley-ridge inflection (VRI) point.

Another particularity occurs in the Diels–Alder addition of reaction 30. Here, the IRC ends on the product side not at a minimum but in another TS structure. Thus, the reaction of the diene with the ethylene molecule proceeds through two TS structures, TS1 and TS2, as depicted in the top panel of Figure 8. From the second TS, which is the boat conformer of cyclohexene, the reaction proceeds further to the two twist boat conformations of the cyclohexene denoted by products 1 and 2 in Figure 8. The reason for this unusual reaction coordinate is the bifurcation of the IRC in TS2. This so-called valley-ridge inflection (VRI) point is a common feature of symmetrical PES as has been shown previously in other Diels–Alder reactions.^65,66^ A schematic two-dimensional description of a model PES with a VRI point is depicted at the bottom of Figure 8. Note the orthogonality of the two IRC paths, which is mandatory for the appearance of a VRI point. In this particular example, the Cremer–Pople coordinates^67^ for the canonical conformers (chair, boat, twist boat and half-chair) of cyclohexene are orthogonal to the Diels–Alder bond forming IRC. To determine the location of the transition states involved in this reaction, we proceeded as follows: First, the saddle method was employed using the diene and ethylene structures in the orientation of Figure 8 as input reactant and the twist boat conformation structure as input product. The Hessian from the resulting structure of the saddle interpolation was calculated and used as the start Hessian for the following local TS optimization with the EF method. Once the TS was optimized, it was characterized by frequency analysis. In this way, the TS1 structure was found. Then, we performed an IRC calculation (forward and reverse) starting from this TS1 structure to verify that it is properly connected to the initial structures of the reaction. Whereas the first IRC calculation led us to the original reactants, the second IRC calculation ended in another PES valley. The frequency analysis of this structure revealed that it corresponds to a transition state (TS2), which is a boat conformation. Thus, a second IRC calculation was performed to verify that the TS2 structure connects two twist conformation structures (Products 1 and Product 2). The obtained results are summarized in Figure 8. The activation energy for the here discussed Diels–Alder cycloaddition is 12.73 kcal/mol, and the corresponding reaction energy is −49.90 kcal/mol.

In general, it is important to highlight that incorporating the LC method into IRC calculations yields smooth IRC paths for all 38 reactions of the test set (see SI). Notable is the fact that all IRC paths converge smoothly into the TS independent from the form of the reaction coordinate. This is remarkable because the positive and negative branches of the IRC paths were calculated separately from each other. Furthermore, the use of the LC approach improves the reliability of IRC end points and, therefore, facilitates the identification of intermediates or TS structures as shown in the case of reaction 30 with the VRI point. A comparison of the LC method with the Gonzalez–Schlegel method for IRC calculations is given in Section S4 of the SI.

## 5 Conclusions

This work presents a reliable and robust algorithm, named the LC method, for the constrained minimization on hyperspheres employing local coordinates in combination with a trust region method. The proposed Levenberg–Marquardt approach guarantees tight convergence to constrained minima on hyperspheres, as demonstrated on the Quapp model surface. Implementation of the LC method into the saddle interpolation of the hierarchical transition state finder results in an efficient double-ended algorithm for the automatic location of (nonintuitive) transition states. These calculations also showed that the LC method works equally well with Newton and quasi-Newton approaches. We demonstrate the reliability of this finder by the automatic transition state optimization for a test set of 38 reactions including dissociation, addition, rearrangement, internal rotation, and ring-opening reactions. Challenging examples in this test set are metal and transition metal cluster rearrangements, reactions with unbalanced activation and reaction energies, as well as reactions with valley-ridge inflection points. Despite these challenges, the hierarchical transition state finder with the LC method in the saddle interpolation worked flawlessly for the 38 test set reactions.

To further study the LC method, we also implement it into the IRC calculation, too. IRC calculations with the LC method yield smooth paths and reliable convergences to reactant and product structures. As a result, reaction intermediates or valley-ridge inflection points can be directly accessed by IRC calculations. This significantly simplifies the calculation of reaction coordinates for complex reactions. The successful validation of the LC method is also a first step toward a robust single-ended transition state finder on the basis of the Dewar methodology. Such work is currently under development in our laboratories.

## Data Availability

All of the data and input information required to reproduce the essential results, including Cartesian molecular transition state structures and IRC plots for the test set reactions, are provided as Supporting Information. The manuscript specifies the software used (http://www.demon-software.com), along with clear descriptions of the fundamental equations. Additionally, detailed settings for all calculations are outlined in Section 3 and the software version is also provided. The deMon2k input and output files for the test set reactions are available in the Zenodo repository (DOI: 10.5281/zenodo.14888501).

## Supporting Information Available

Input and output files for the test set reactions (ZIP)
Cartesian coordinates of the optimized transition state structures and the IRC plots of the test set reactions (PDF)
