# Supplementary material for: Constrained Structure Minimizations on Hyperspheres for Minimum Energy Path Following
Source: J Chem Inf Model. 2025 Apr 1;65(7):3488–501. doi: 10.1021/acs.jcim.4c02351 (PMC12004539; doi:10.1021/acs.jcim.4c02351)
Supplement: Supplementary file 2 — ci4c02351_si_002.pdf [file ci4c02351_si_002.pdf]

Supplementary Material

Constrained Structure Minimizations on Hyperspheres for Minimum Energy Path Following

Jorge Alberto Sanchez Alvarez*, Luis López-Sosa,

Andreas M. Köster, Patrizia Calaminici*

Chemistry Department, CINVESTAV, Av. Instituto Politécnico Nacional 2508,

Col. San Pedro Zacatenco, Del. Gustavo A. Madero, C.P. 07360, Mexico City, Mexico

S1. Rotation Algorithm

For the transformation from the global to the local coordinate system, we describe in the following the construction of the **U** matrix for the first rotation of the **c** vector. This first rotation is given by:

$$\mathbf{c}^{(1)} = \mathbf{U}_1\mathbf{c} = \begin{pmatrix} \cos\phi_1 & -\sin\phi_1 & 0 \\ \sin\phi_1 & \cos\phi_1 & 0 \\ 0 & 0 & \mathbf{I} \end{pmatrix} \begin{pmatrix} c_1 \\ c_2 \\ \vdots \\ c_n \end{pmatrix} = \begin{pmatrix} c_1^{(1)} \\ 0 \\ \vdots \\ c_n \end{pmatrix} \tag{A1}$$

This initial transformation, denoted as the first rotation, plays a pivotal role where we can choose ϕ_1 such that the first component of the vector $\mathbf{c}^{(1)}$ lies on the first axis and the second component is zeroed out. The global coordinates are rotated counter-clockwise through the angle ϕ_1 about the origin. Note that this rotation is independent of the orientation of the coordinate system. Therefore, the first two components of $\mathbf{c}^{(1)}$ are:

$$c_1^{(1)} = c_1\cos\phi_1 - c_2\sin\phi_1 \tag{A2}$$

$$0 = c_1\sin\phi_1 + c_2\cos\phi_1 \tag{A3}$$

From Eq. (A3) the angle ϕ_1 is determined by solving the following equation:

$$c_1 \sin \phi_1 \equiv -c_2 \cos \phi_1 \tag{A4}$$

To solve equation (A4) while maintaining a counter-clockwise rotation orientation, we consider the following three cases:

Case 1: c_1=0

$$c_2 \cos \phi_1 = 0 \quad \Rightarrow \quad \phi_1 = \frac{\pi}{2} \tag{A5}$$

Case 2: c_2=0

$$c_1 \sin \phi_1 = 0 \quad \Rightarrow \quad \phi_1 = 0 \tag{A6}$$

Case 3: $c_1 \neq 0 \quad \wedge c_2 \neq 0$

$$\tan \phi_1 = -\frac{c_2}{c_1} \quad \Rightarrow \quad \phi_1 = -\arctan\left(\frac{c_2}{c_1}\right) \tag{A7}$$

In this third case we have to consider that in order to keep the counter-clockwise rotation of the axis and the $\mathring{\mathbf{e}}_1$ vector pointing outwards of the hypersphere, it is necessary to consider the sign of the ϕ_1 angle. In the case $\phi_1 < 0$, the axis is rotated in the clockwise direction and the $\mathring{\mathbf{e}}_1$ vector does not point outwards of the hypersphere. To resolve this remedy it is sufficient to invert the sign of all trigonometric functions in $\mathbf{U}_1$.

S2. Cartesian coordinates of optimized transition states

Optimized transition state coordinates for the test set reactions of Figure 4 of the article. The transition state structures from reaction 1 to 37 were fully optimized and characterized by frequency analysis using the PBE/DZVP-GGA/GEN-A2* level of theory. The Zr atom of reaction 36 was treated with ECP. The M06 functional was used for reaction 38. All coordinates are given in Å. First frequencies are also given in cm^{-1}.

TS-RXN1

ω = 996.3i

Si	−0.087 142	0.062 845	0.000 005
H	−0.195 077	−0.824 406	1.212 163
H	−0.195 392	−0.824 475	−1.212 059
H	1.387 392	0.524 307	−0.000 141
H	1.431 218	−0.626 557	−0.000 108

TS-RXN2

ω = 1578.2i

N	−0.496 241	−0.116 586	0.000 922
C	0.727 388	−0.031 064	−0.000 569
H	−1.526 007	−0.142 341	−0.006 519
H	1.326 126	0.897 330	0.000 729
H	−1.571 990	1.235 312	−0.000 242

TS-RXN3

$\omega = 2209.5i$

C	−0.551 134	0.111 549	0.000 013
N	0.689 802	0.004 042	−0.000 002
H	−1.385 449	0.842 052	−0.000 079
H	−0.259 137	−1.076 101	−0.000 058
H	−1.373 660	−1.151 386	0.000 011

TS-RXN4

$\omega = 531.2i$

C	1.503 298	0.148 715	0.001 569
N	0.997 362	1.263 629	−0.000 902
H	2.541 208	−0.187 130	0.003 855
N	0.591 634	−1.377 028	−0.000 670
N	−0.591 005	−1.377 322	−0.000 796
C	−1.503 378	0.148 089	0.001 600
N	−0.997 929	1.263 221	−0.000 930
H	−2.541 125	−0.188 260	0.004 200

TS-RXN5

$\omega = 1867.8i$

C	−0.792 950	−0.000 005	−0.023 762
C	0.665 412	−0.000 035	−0.118 591
H	−1.316 348	0.940 742	0.164 999
H	−1.316 364	−0.940 648	0.165 471
H	1.154 106	−0.921 518	−0.453 651
H	1.154 129	0.921 232	−0.454 214
H	0.315 907	0.000 309	1.052 778
H	1.528 355	0.000 362	1.220 944

TS-RXN6

$\omega = 1619.1i$

C	1.193 154	−0.459 960	−0.000 002
C	0.453 093	0.747 101	0.000 003
H	1.656 992	−0.793 530	0.934 530
H	0.238 873	1.286 084	0.929 211
H	1.657 041	−0.793 505	−0.934 515
H	0.238 939	1.286 182	−0.929 154
H	−0.097 793	−0.815 751	−0.000 005
F	−1.236 759	−0.190 526	−0.000 004

TS-RXN7

$\omega = 1129.2i$

C	1.069 231	−0.503 862	0.000 029
O	1.898 610	0.309 933	−0.000 011
H	0.143 194	−1.166 722	−0.000 192
Cl	−1.223 137	0.064 004	0.000 000

TS-RXN8

$\omega = 1371.3i$

P	0.208 845	0.010 153	−0.081 576
O	1.058 582	1.180 800	0.392 874
O	0.893 479	−1.349 361	−0.035 693
O	−0.833 287	0.285 614	−1.229 682
H	−1.641 139	0.081 289	−0.201 061
O	−1.326 099	−0.079 983	0.960 150
H	−1.485 687	−0.981 708	1.316 593

TS-RXN9

ω = 1061.7i

C	−1.890 946	−0.414 399	0.005 741
C	−0.831 400	0.679 222	−0.031 536
H	−1.712 380	−1.174 758	−0.773 222
H	−0.701 152	1.130 592	−1.024 411
H	−1.918 884	−0.935 447	0.979 946
H	−1.000 889	1.483 393	0.703 700
H	−2.896 127	0.014 703	−0.166 303
Si	1.353 755	−0.141 314	0.043 462
H	1.807 722	1.186 886	−0.502 947
H	0.936 497	−0.985 346	−1.130 857
H	0.204 348	0.061 851	1.010 436

TS-RXN10

ω = 903.3i

C	−0.663 884	−0.512 578	0.000 079
O	−1.441 601	0.322 312	−0.000 048
H	0.205 684	−1.195 211	−0.001 150
N	1.748 517	0.099 865	0.000 066
H	2.510 776	−0.587 008	−0.105 653
H	1.851 202	0.775 717	−0.771 244
H	1.928 410	0.610 642	0.876 961

TS-RXN11

ω = 819.8i

C	−1.307 094	0.236 188	−0.344 086
C	−0.547 737	1.234 004	0.273 514
C	−1.209 711	−1.105 709	0.156 211
H	−0.527 709	2.248 206	−0.144 810
H	−0.411 624	1.186 977	1.361 699
H	−1.734 851	−1.899 026	−0.394 537
H	−1.604 123	0.381 473	−1.393 017
H	−1.283 796	−1.231 035	1.248 753
H	0.052 165	−1.261 506	0.033 187
C	1.519 106	−0.839 823	−0.002 920
C	1.427 921	0.566 364	−0.112 082
H	1.806 923	1.186 763	0.707 984
H	1.519 807	1.024 641	−1.103 221
H	1.715 275	−1.435 926	−0.903 607
H	1.868 291	−1.285 238	0.937 467

TS-RXN12

$\omega = 1990.1i$

C	0.026 322	−0.541 652	−0.035 423
O	1.063 404	0.242 951	0.014 212
C	−1.175 368	0.213 709	0.033 633
H	−2.116 267	−0.207 569	0.410 812
H	−1.309 123	0.864 909	−0.847 384
H	0.183 880	−1.634 445	−0.078 916
H	0.054 173	1.028 550	0.311 216

TS-RXN13

$\omega = 301.7i$

C	−2.028 743	0.110 066	−0.138 243
C	−1.321 732	1.323 698	−0.040 880
O	−1.550 441	−1.062 967	−0.236 421
H	−1.850 271	2.256 264	0.187 412
H	−0.346 610	1.392 401	−0.531 391
H	−3.144 200	0.198 711	−0.187 650
C	0.699 255	−0.556 822	0.737 862
C	1.764 694	0.304 200	0.362 325
C	2.673 024	−0.011 053	−0.623 951
H	0.621 631	−1.552 088	0.295 761
H	0.089 597	−0.338 469	1.617 759
H	3.514 396	0.648 629	−0.860 333
H	1.864 674	1.260 072	0.895 705
H	2.572 902	−0.935 875	−1.204 987

TS-RXN14

$\omega = 189.5i$

C	0.677 113	−0.311 821	−0.370 852
C	1.727 820	0.136 662	0.347 019
C	−0.677 133	0.311 747	−0.370 947
C	−1.727 802	−0.136 585	0.347 074
H	−2.702 467	0.362 652	0.299 146
H	2.702 437	−0.362 683	0.299 234
H	1.638 533	1.008 078	1.007 772
H	−1.638 418	−1.007 801	1.008 073
H	−0.814 780	1.188 727	−1.023 045
H	0.814 727	−1.189 007	−1.022 688

TS-RXN15

$\omega = 239.0i$

C	0.683 080	0.293 916	0.355 219
O	1.576 887	−0.331 469	−0.206 448
C	−0.701 272	0.462 993	−0.209 841
C	−1.722 768	−0.371 572	0.061 441
H	0.869 333	0.797 431	1.344 129
H	−2.713 484	−0.219 428	−0.381 186
H	−1.597 594	−1.237 501	0.723 128
H	−0.842 816	1.329 212	−0.873 591

TS-RXN16

$\omega = 684.4i$

C	1.910 033	−0.115 284	0.279 849
H	1.898 749	−0.051 886	1.388 399
H	2.971 256	−0.216 889	−0.011 096
C	1.345 353	1.193 594	−0.310 710
H	2.008 065	2.042 304	−0.055 535
H	1.292 469	1.113 686	−1.410 430
C	1.133 628	−1.363 720	−0.184 136
H	1.594 904	−2.271 473	0.246 239
H	1.220 364	−1.442 067	−1.283 955
C	−0.055 944	1.485 771	0.229 918
H	−0.658 847	2.211 426	−0.328 685
H	0.047 693	1.905 495	1.277 198
C	−0.365 947	−1.322 193	0.182 071
H	−0.899 463	−2.126 848	−0.357 694
H	−0.516 655	−1.530 169	1.266 969
C	−0.974 849	−0.018 740	−0.368 514
F	−2.336 788	0.080 204	0.027 794
H	−0.570 110	0.529 783	0.788 633

TS-RXN17

$\omega = 276.6i$

C	−0.695 749	−0.851 237	0.082 061
C	−1.048 177	0.509 322	−0.275 964
C	0.272 233	0.233 211	0.561 137
C	1.400 083	0.049 727	−0.272 121
H	2.396 024	−0.173 631	0.131 473
H	1.264 037	−0.013 805	−1.355 844
H	−0.416 457	−1.644 909	−0.626 359
H	−0.973 910	0.950 403	−1.285 737
H	−1.748 225	1.025 831	0.397 012
H	0.331 854	0.558 910	1.606 061

TS-RXN18

$\omega = 143.1\text{i}$

C	−1.468 012	−0.514 530	1.495 189
C	−1.830 009	−0.453 641	0.059 457
H	−1.278 706	0.502 540	1.882 909
C	−1.342 926	−1.447 136	−0.931 295
C	−3.056 647	0.314 773	−0.304 415
H	−0.478 590	−2.021 522	−0.575 245
H	−3.209 482	1.193 629	0.336 058
H	−0.595 465	−1.149 603	1.694 257
H	−1.074 104	−0.958 219	−1.883 507
H	−3.074 277	0.602 331	−1.366 264
H	−3.906 633	−0.387 350	−0.144 641
H	−2.371 376	−0.871 406	2.042 179
H	−2.209 062	−2.106 689	−1.173 608
O	−0.780 283	2.006 744	−0.163 209
H	−0.652 566	2.550 388	−0.964 801
H	0.082 101	1.486 197	−0.088 872
Br	1.550 073	−0.071 439	−0.012 215

TS-RXN19

$\omega = 748.9\text{i}$

C	−1.830 447	−0.272 579	0.003 355
C	−2.166 253	1.092 919	0.104 379
C	−1.775 192	−1.137 386	1.227 165
C	−1.788 281	−0.945 916	−1.335 619
H	−1.056 277	−1.961 684	1.118 219
H	−1.049 555	−1.759 672	−1.362 524
H	−2.564 322	1.435 952	1.071 385
H	−1.535 157	−0.559 389	2.132 729
H	−1.580 672	−0.235 007	−2.150 046
H	−2.613 929	1.560 000	−0.786 387
H	−2.794 654	−1.381 493	−1.515 712
H	−2.788 827	−1.572 113	1.364 712
H	−1.009 718	1.739 519	0.066 032
O	0.122 666	2.409 215	−0.047 469
H	0.324 188	2.786 307	0.834 116
H	0.722 198	1.526 160	−0.094 841
Br	1.313 026	−0.312 471	0.001 064

TS-RXN20

ω = 123.5i

O	−1.231 427	−1.429 465	−0.185 608
C	−0.302 813	−0.560 996	−0.072 909
Li	−2.479 542	−0.154 413	−0.054 393
C	0.510 902	−0.048 891	−1.270 218
C	0.535 316	−0.373 717	1.202 242
C	1.692 723	0.716 984	−0.651 797
C	1.948 207	−0.020 929	0.685 879
C	−1.743 772	1.683 392	0.290 412
H	−0.099 082	0.529 954	−1.979 888
H	0.485 085	−1.290 615	1.813 900
H	0.119 220	0.454 740	1.799 715
H	−0.806 125	2.186 186	−0.000 173
H	2.528 284	0.583 562	1.403 178
H	2.582 461	0.735 223	−1.303 471
H	1.402 872	1.764 891	−0.454 467
H	0.865 120	−0.957 547	−1.800 550
H	2.521 605	−0.947 581	0.495 069
H	−2.566 338	2.232 487	−0.224 387
H	−1.877 264	1.829 064	1.383 930

TS-RXN21

ω = 1619.9i

C	−0.616 454	−0.046 033	−0.000 025
C	0.740 791	0.080 971	0.000 004
H	−1.509 945	0.676 840	0.000 144
H	0.028 302	−1.093 171	0.000 105

TS-RXN22

ω = 1402.8i

C	−0.811 425	0.057 915	0.000 004
H	−1.240 317	−0.438 000	0.908 958
H	−1.240 345	−0.437 822	−0.909 028
O	0.762 020	−0.047 730	−0.000 007
H	0.054 090	0.943 317	0.000 131

TS-RXN23

ω = 888.4i

C	1.125 530	−0.164 430	0.000 177
O	1.886 850	0.738 629	0.002 581
O	0.399 481	−1.122 477	−0.002 103
H	2.126 175	−1.883 659	0.001 758
C	−1.153 306	0.779 410	−0.005 116
C	−2.367 497	−0.086 584	0.004 108
H	−0.868 811	1.299 778	0.918 216
H	−0.873 122	1.286 247	−0.937 233
H	−2.426 816	−0.731 895	−0.888 782
H	−2.421 796	−0.719 920	0.905 851
H	−3.284 417	0.545 844	0.002 495

TS-RXN24

ω = 1567.0i

N	−0.728 271	−0.043 039	0.048 805
C	0.840 300	−0.066 722	0.044 699
H	−1.046 016	0.918 607	−0.213 578
H	1.182 955	0.995 656	−0.170 598
H	−0.029 929	−0.521 087	−0.826 696

TS-RXN25

ω = 997.7i

C	−0.067 612	−0.695 167	0.135 931
C	−0.982 991	0.345 124	−0.022 946
C	1.050 962	0.249 407	−0.046 717
H	1.433 660	0.868 454	0.774 549
H	−0.153 893	−1.660 753	−0.373 091
H	1.466 269	0.424 386	−1.046 773
H	−0.943 643	1.236 890	0.616 431
H	−1.806 664	0.330 239	−0.760 790

TS-RXN26

ω = 35.4i

Na	0.901 049	0.007 409	−1.092 584
Na	2.296 074	2.776 456	0.175 374
Na	2.340 844	−2.739 659	0.173 666
Na	−1.071 639	2.218 758	0.571 886
Na	−1.034 310	−2.234 357	0.574 408
Na	−3.432 019	−0.028 607	−0.402 750

TS-RXN27

$\omega = 27.8i$

Na	−0.301 388	1.790 524	0.000 089
Na	2.047 033	0.728 279	−2.314 300
Na	2.047 212	0.728 396	2.314 132
Na	1.675 012	−1.775 772	0.000 005
Na	−3.469 162	0.379 943	0.000 132
Na	−0.999 437	−0.925 589	−2.086 708
Na	−0.999 270	−0.925 781	2.086 651

TS-RX28

$\omega = 326.0i$

N	1.701 111	0.043 758	0.018 244
O	2.541 250	−0.610 837	0.684 444
O	1.643 322	0.043 648	−1.242 418
C	0.740 679	0.828 313	0.738 351
C	−0.085 715	1.713 909	0.048 420
H	0.834 535	0.744 283	1.822 082
H	0.179 658	1.949 418	−0.986 253
H	−0.567 918	2.509 650	0.626 383
C	−1.886 059	0.707 789	−0.360 059
C	−1.450 292	−0.297 509	−1.250 464
C	−0.940 350	−1.399 561	−0.499 865
C	−1.120 841	−1.146 092	0.854 184
C	−2.012 991	0.055 159	1.007 367
H	−0.414 143	−2.258 633	−0.924 730
H	−0.818 763	−1.801 755	1.675 912
H	−1.399 197	−0.201 782	−2.338 753
H	−2.512 636	1.551 506	−0.670 932
H	−1.801 128	0.698 369	1.875 975
H	−3.061 026	−0.301 403	1.113 500

TS-RXN29

$\omega = 352.5i$

C	1.221 551	1.149 839	−0.122 553
O	1.614 565	2.264 940	0.165 596
O	1.707 376	0.000 225	0.546 738
C	1.221 941	−1.149 619	−0.122 537
O	1.615 434	−2.264 560	0.165 616
C	0.234 500	0.702 014	−1.135 330
C	0.234 744	−0.702 130	−1.135 303
H	−0.036 092	−1.362 055	−1.961 740
H	−0.036 380	1.361 770	−1.961 891
C	−1.714 290	1.163 937	−0.052 082
C	−1.374 699	0.708 012	1.238 639
C	−1.374 616	−0.708 070	1.238 790
C	−1.714 323	−1.164 349	−0.051 753
C	−2.339 228	−0.000 328	−0.787 715
H	−1.045 460	1.342 894	2.066 136
H	−1.045 301	−1.342 721	2.066 432
H	−1.882 157	2.213 592	−0.312 811
H	−1.881 950	−2.214 092	−0.312 276
H	−2.234 068	−0.000 483	−1.883 343
H	−3.426 925	−0.000 252	−0.556 162

TS-RXN30

$\omega = 412.9i$

C	−1.299 372	0.322 826	−0.711 272
C	−0.452 553	−0.493 133	−1.454 782
C	−1.299 340	0.322 820	0.711 307
C	−0.452 492	−0.493 142	1.454 792
H	−0.122 813	−1.461 243	1.065 809
H	−0.398 918	−0.383 636	2.544 382
H	−0.399 042	−0.383 666	−2.544 379
H	−0.122 890	−1.461 232	−1.065 784
H	−1.832 035	1.135 239	1.223 589
H	−1.832 098	1.135 251	−1.223 514
C	1.643 762	0.188 860	0.696 595
C	1.643 767	0.188 847	−0.696 640
H	1.536 400	1.127 240	−1.248 729
H	1.536 416	1.127 268	1.248 663
H	2.105 806	−0.638 539	−1.247 260
H	2.105 825	−0.638 508	1.247 225

TS-RXN31

$\omega = 346.0\text{i}$

C	1.712 608	0.287 477	0.512 644
O	2.101 568	1.406 703	0.864 081
O	2.376 832	−0.463 222	−0.436 274
C	0.491 256	−0.368 341	1.008 985
C	0.201 173	−1.739 917	0.854 358
C	3.587 645	0.144 495	−0.939 601
H	0.020 538	0.194 435	1.824 129
H	0.891 103	−2.370 502	0.286 172
H	−0.384 863	−2.248 574	1.626 107
H	3.976 058	−0.557 561	−1.692 171
H	4.321 379	0.284 713	−0.127 635
H	3.377 333	1.126 477	−1.396 536
O	−1.249 903	−1.845 358	−0.466 962
N	−1.688 271	−0.609 836	−0.526 236
C	−0.816 110	0.406 192	−0.697 824
C	−2.845 429	−0.178 840	0.303 846
C	−1.462 664	1.727 951	−0.380 556
C	−2.916 520	1.331 839	0.023 462
H	0.010 711	0.259 285	−1.396 994
H	−1.440 510	2.422 010	−1.238 856
H	−3.739 869	−0.745 984	0.003 836
H	−3.282 364	1.897 643	0.894 345
H	−3.604 036	1.526 385	−0.816 511
H	−0.914 614	2.223 921	0.441 583
H	−2.611 814	−0.417 468	1.360 366

TS-RXN32

$\omega = 332.4i$

C	2.306 964	0.548 846	0.017 307
O	2.853 998	1.614 950	0.273 410
C	2.399 202	−0.804 546	0.270 440
C	1.415 620	−1.886 192	−0.081 026
C	3.741 756	−1.263 119	0.812 709
H	0.386 771	−1.522 195	−0.212 114
H	4.394 222	−0.412 098	1.060 996
H	4.274 765	−1.911 616	0.088 286
H	1.390 932	−2.634 716	0.733 656
H	1.711 666	−2.439 131	−0.997 681
H	3.589 820	−1.866 067	1.730 071
C	1.004 965	0.850 582	−1.430 502
C	0.009 694	1.249 562	−0.531 971
C	−1.245 085	0.577 682	−0.223 239
C	−1.791 790	−0.456 282	−1.034 295
C	−3.017 280	−1.051 499	−0.708 038
C	−3.733 830	−0.629 418	0.430 425
C	−3.215 779	0.403 076	1.236 909
C	−1.990 569	1.000 310	0.911 693
H	1.672 384	1.604 756	−1.857 941
H	−1.263 061	−0.773 724	−1.940 599
H	−3.770 853	0.741 205	2.119 316
H	−3.425 248	−1.842 270	−1.348 106
H	−4.693 400	−1.095 742	0.680 675
H	−1.586 894	1.801 643	1.542 667
H	0.202 942	2.171 484	0.033 304
H	0.862 804	−0.046 386	−2.044 969

TS-RXN33

$\omega = 5.0i$

C	0.258 432	1.469 535	0.581 234
O	0.273 533	2.704 966	0.692 789
C	−0.430 973	0.637 592	1.598 943
C	−0.404 694	−0.723 773	1.588 446
C	0.309 254	−1.514 566	0.555 457
O	0.360 515	−2.751 158	0.642 848
C	0.975 970	−0.781 328	−0.599 907
C	0.947 247	0.778 786	−0.587 564
H	−0.940 506	1.197 856	2.393 736
H	−0.891 261	−1.314 748	2.375 589
H	0.480 168	−1.162 797	−1.511 579
H	0.434 366	1.155 686	−1.491 915
C	2.480 941	1.161 596	−0.699 215
C	3.130 556	0.709 648	0.603 986
C	3.155 467	−0.646 582	0.595 289
C	2.523 009	−1.105 455	−0.713 795
H	3.404 227	1.372 828	1.430 260
H	3.453 605	−1.309 690	1.413 138
C	2.962 183	0.042 780	−1.655 556
H	2.643 028	2.207 526	−0.995 491
H	2.723 790	−2.140 665	−1.023 694
H	4.054 060	0.064 069	−1.808 121
H	2.443 596	0.039 231	−2.631 871
C	−3.532 168	1.127 639	−0.086 258
C	−2.882 335	0.883 169	−1.265 144
C	−2.793 518	−0.568 591	−1.477 045
C	−3.387 164	−1.215 274	−0.427 751
C	−3.922 970	−0.187 877	0.536 263
H	−3.482 032	−2.296 269	−0.294 738
H	−3.754 697	2.107 097	0.345 524
H	−2.490 655	1.642 856	−1.949 773
H	−2.329 388	−1.047 786	−2.345 631
H	−3.510 490	−0.311 470	1.558 012
H	−5.024 204	−0.272 444	0.645 461

TS-RXN34

$\omega = 1930.7\mathrm{i}$

C	−0.502 345	0.917 274	0.059 531
O	0.286 876	1.780 843	0.656 058
C	−1.777 525	1.150 038	−0.520 096
C	−2.682 910	0.089 131	−0.411 793
C	−2.321 527	−1.180 715	0.142 675
C	−0.997 265	−1.453 503	0.473 419
C	0.011 977	−0.434 628	0.322 808
H	−3.089 511	−1.954 406	0.247 346
H	−2.084 583	2.151 091	−0.840 973
H	−3.725 503	0.252 490	−0.712 583
H	−0.698 418	−2.470 756	0.760 871
C	1.318 389	−0.916 184	−0.393 071
C	2.476 788	0.047 817	−0.446 639
C	3.715 425	−0.237 679	0.002 580
H	4.535 404	0.483 550	−0.087 545
H	2.287 333	1.027 129	−0.907 311
H	3.946 939	−1.201 301	0.475 759
H	1.040 612	−1.199 362	−1.429 121
H	1.636 420	−1.843 622	0.117 089
H	0.642 072	0.539 833	1.145 245

TS-RXN35

$\omega = 289.4$i

C	0.655 422	−1.989 907	−0.024 733
O	1.631 939	−2.633 210	0.019 872
Co	0.095 504	0.120 793	0.083 939
C	1.268 486	0.326 409	1.406 103
C	1.639 335	1.480 533	0.677 963
C	1.381 163	0.429 646	−1.302 128
C	2.116 456	1.411 029	−0.722 697
H	1.388 908	0.081 355	−2.343 676
H	1.243 279	0.169 396	2.494 238
H	1.495 545	2.464 790	1.162 639
H	2.863 495	2.096 705	−1.149 837
C	−2.250 783	−0.730 490	0.663 067
C	−2.164 323	−0.837 831	−0.737 066
C	−1.672 714	0.418 194	−1.254 331
C	−1.570 097	1.340 196	−0.164 404
C	−1.837 335	0.597 828	1.044 201
H	−2.297 391	−1.750 786	−1.321 222
H	−2.497 101	−1.538 962	1.355 376
H	−1.511 345	0.662 582	−2.306 255
H	−1.853 631	1.008 199	2.056 348
H	−1.311 054	2.399 156	−0.237 206

TS-RXN36

$\omega = 165.4$i

C	−0.082 378	1.479 929	−1.677 973
Zr	0.012 073	−0.029 621	−0.074 630
C	−0.163 023	1.655 559	1.462 353
Cl	2.178 537	−0.925 890	0.116 145
Cl	−1.964 371	−1.293 513	0.086 066
H	0.812 367	1.661 215	1.973 611
H	−1.037 826	1.352 629	2.060 529
H	−0.898 297	2.071 754	−2.116 570
H	−0.202 67	0.443 301	−2.161 108
H	0.903 460	1.865 415	−1.989 119
C	−0.364 143	2.517 364	0.363 853
H	0.442 147	3.176 002	0.023 807
H	−1.381 228	2.812 971	0.084 467

TS-RXN37

ω = 46.4i

V	0.264 392	−0.025 923	−0.081 396
Cu	1.493 168	1.122 361	1.972 644
Cu	0.053 595	2.519 698	0.634 179
Cu	−1.837 694	1.469 531	−0.474 857
Cu	−2.381 744	−0.863 951	−0.690 756
Cu	−1.061 165	−1.966 943	0.987 639
Cu	0.900 979	−1.202 427	2.196 675
Cu	2.776 115	−0.539 701	0.538 090
Cu	2.273 473	1.634 957	−0.401 775
Cu	0.338 274	1.976 269	−1.795 877
Cu	−0.884 620	0.048 528	−2.470 462
Cu	−0.406 412	−2.127 646	−1.542 351
Cu	1.358 766	−2.419 064	0.035 853
Cu	1.699 679	−0.485 977	−1.943 921
Cu	−1.081 312	0.501 865	1.892 205
Cu	−3.453 051	0.353 283	1.127 965

TS-RXN38

ω = 168.3i

C	2.095 732	−0.012 065	−0.000 562
O	2.498 304	1.101 440	0.001 406
O	2.147 177	−1.199 901	−0.004 570
C	−0.154 342	0.208 714	0.005 263
C	−1.177 259	1.158 275	−0.002 784
N	−2.411 398	0.574 507	0.019 670
N	−2.222 002	−0.764 743	−0.022 643
C	−0.878 724	−0.985 363	0.006 985
H	−3.002 847	−1.395 726	0.117 553
H	−3.333 710	0.964 955	−0.138 133
H	−1.132 914	2.243 939	−0.021 739
H	−0.516 810	−2.010 489	0.027 781

S3. IRC plots of test set reactions

In Figure S1 are presented the IRC plots of the test set reactions of Figure 4 of the article.

rxn1

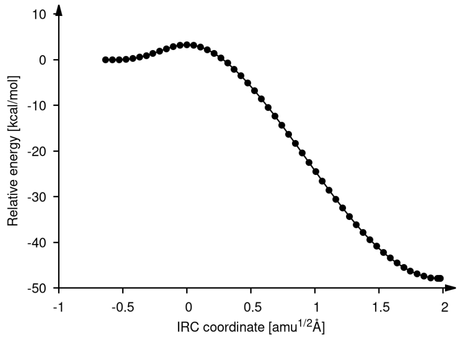

rxn20

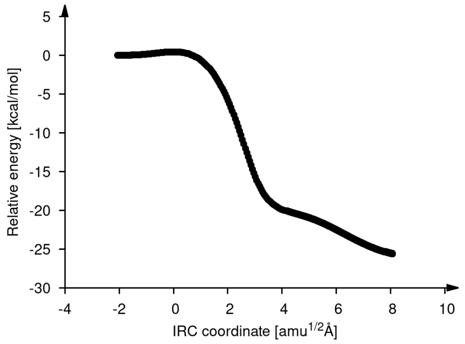

rxn2

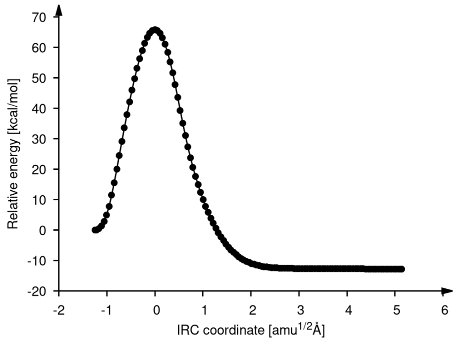

rxn21

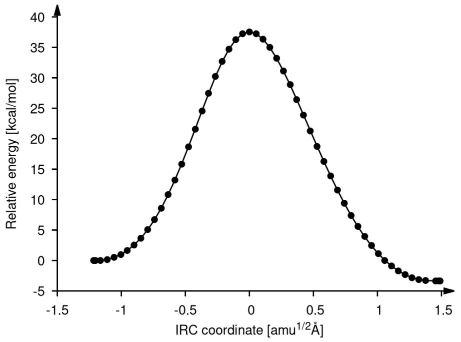

rxn3

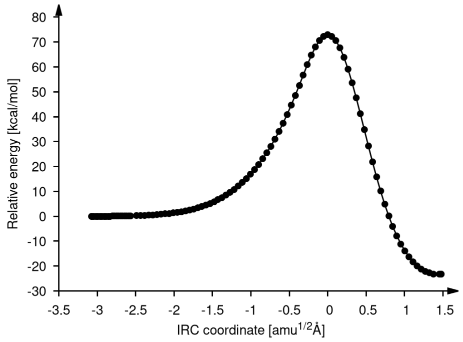

rxn22

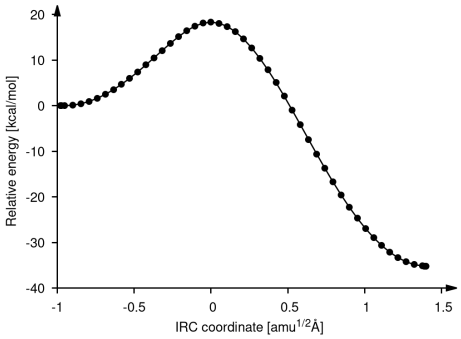

rxn4

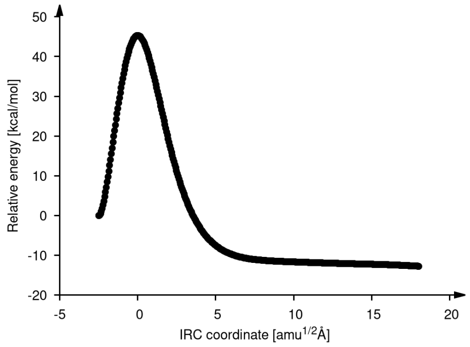

rxn23

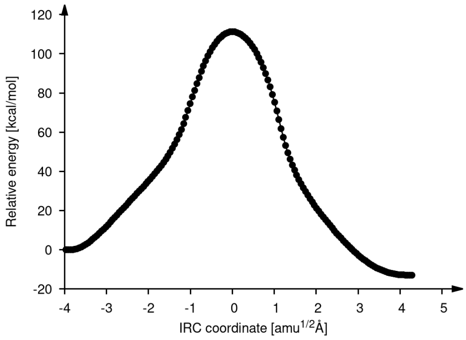

Figure S1: Continued on next page...

rxn5

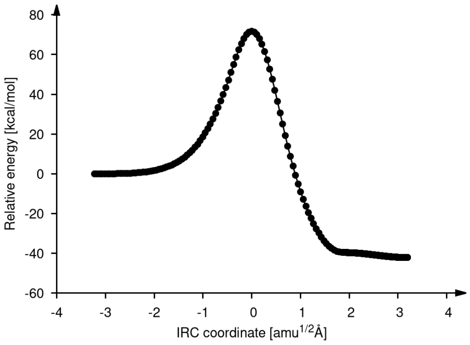

rxn6

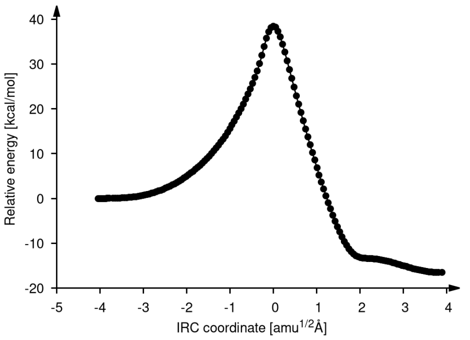

rxn7

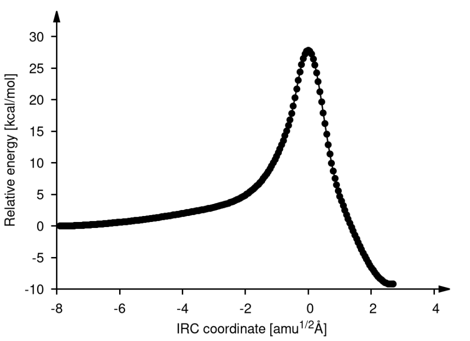

rxn8

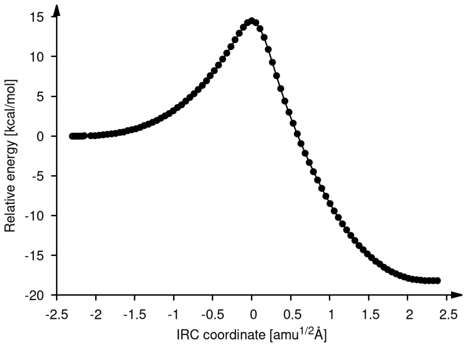

rxn24

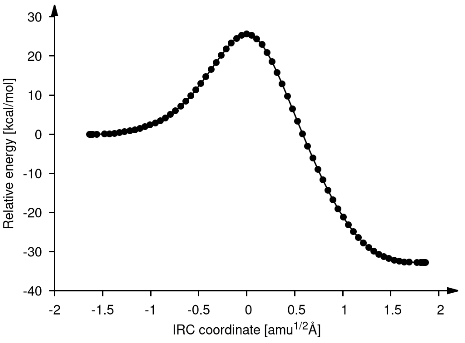

rxn25

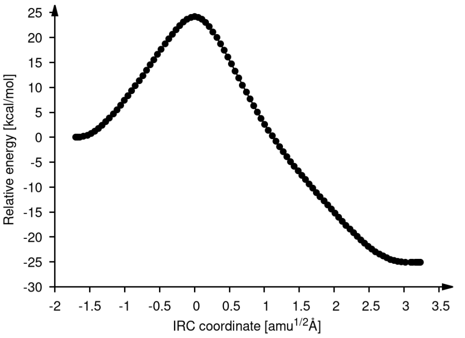

rxn26

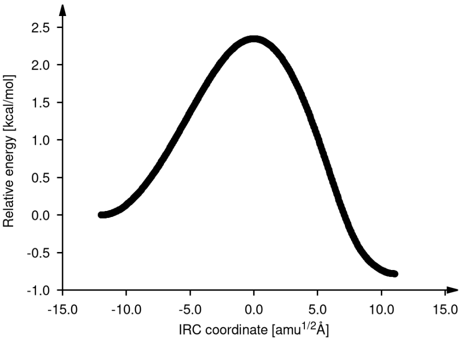

rxn27

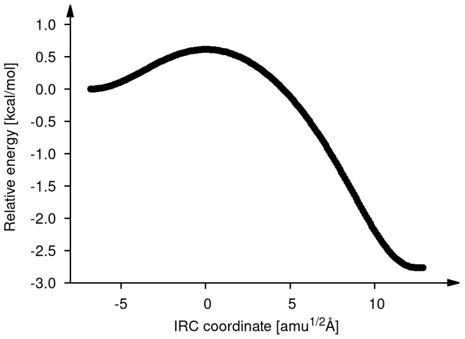

Figure S1: Continued on next page...

rxn9

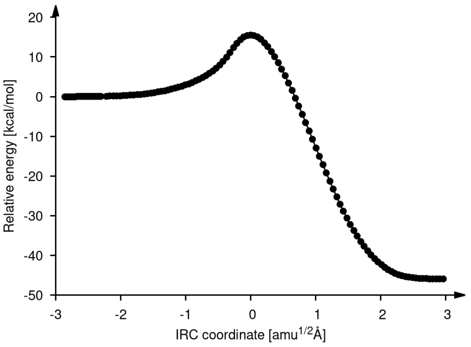

rxn28

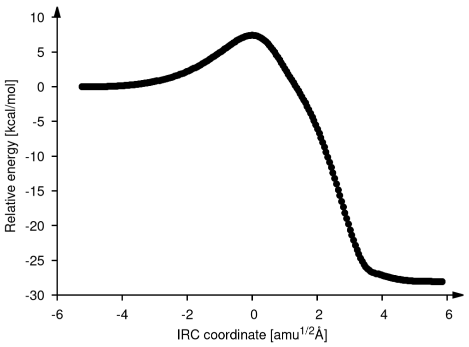

rxn10

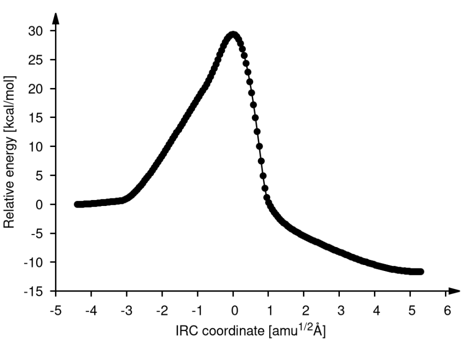

rxn29

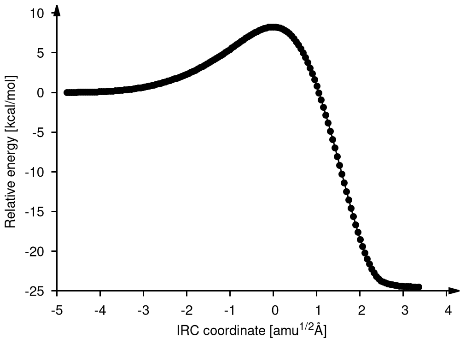

rxn11

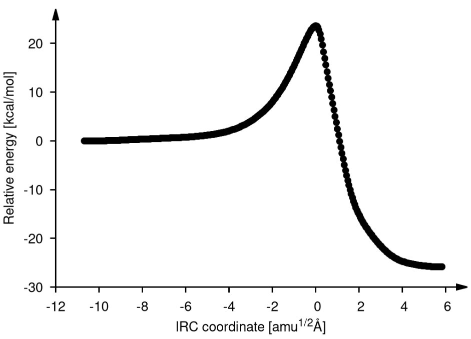

rxn30

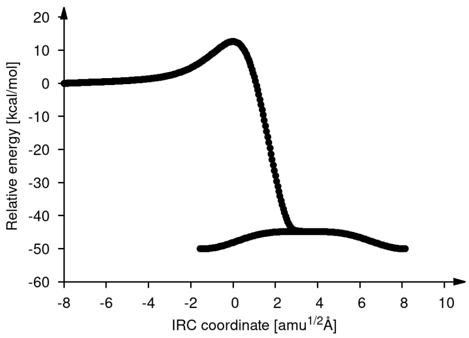

rxn12

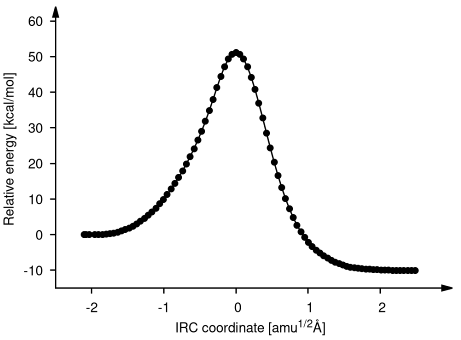

rxn31

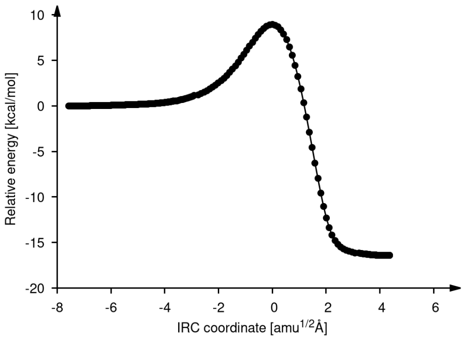

Figure S1: Continued on next page...

rxn13

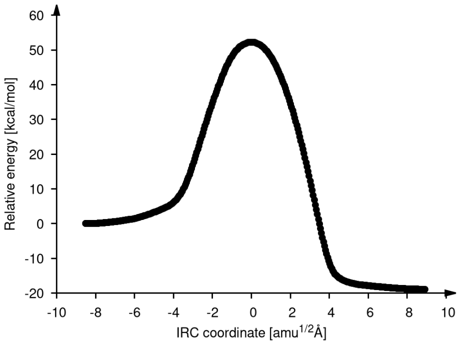

rxn32

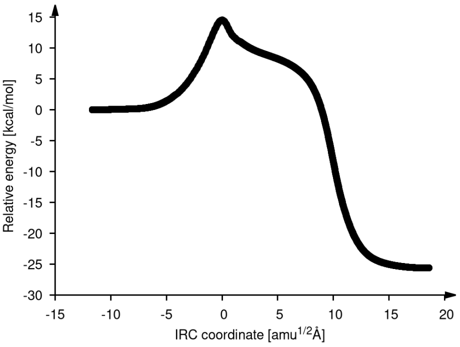

rxn14

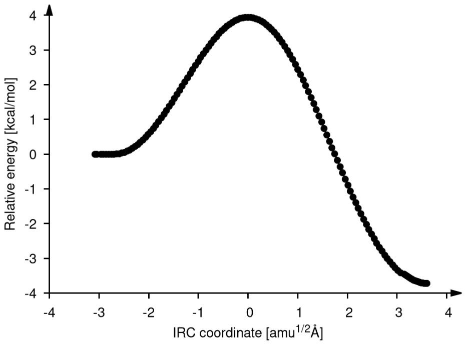

rxn33

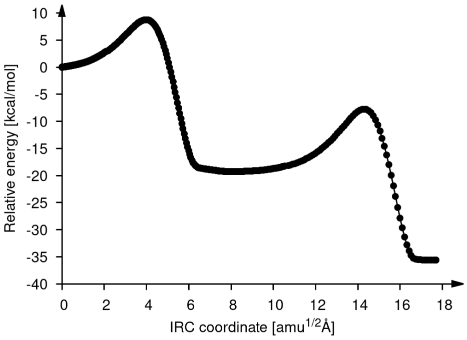

rxn15

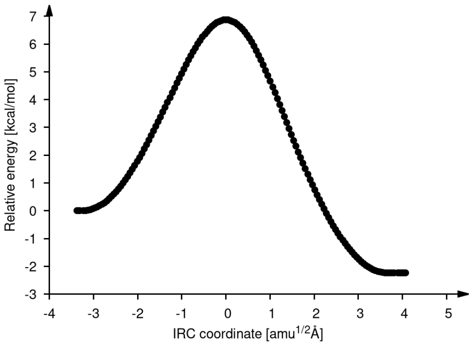

rxn34

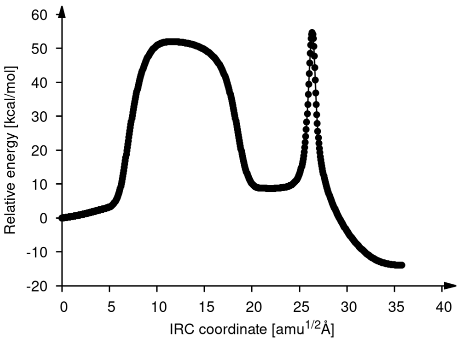

rxn16

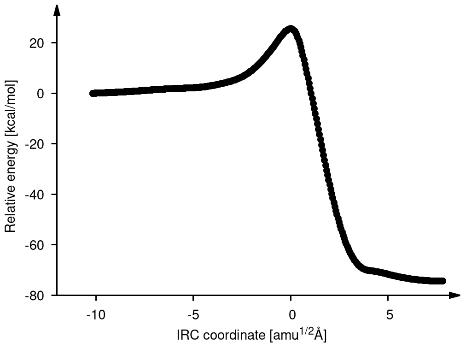

rxn35

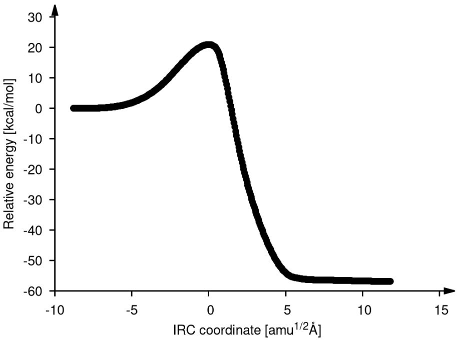

Figure S1: Continued on next page...

rxn17

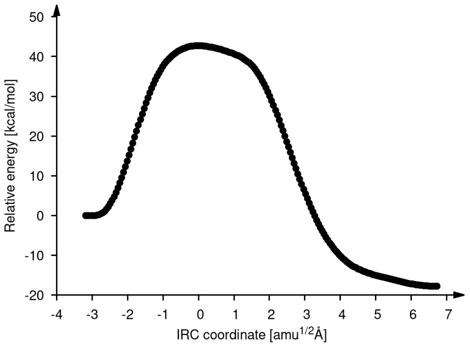

rxn36

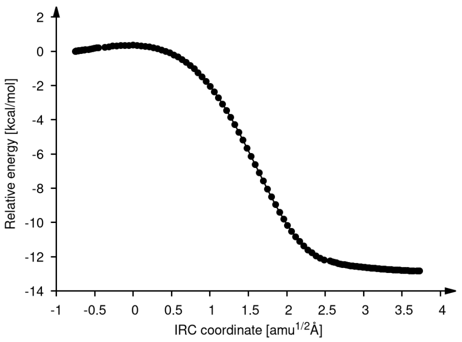

rxn18

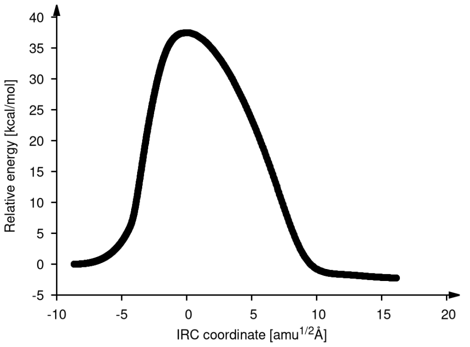

rxn37

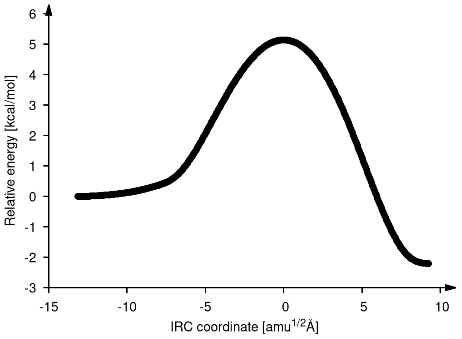

rxn19

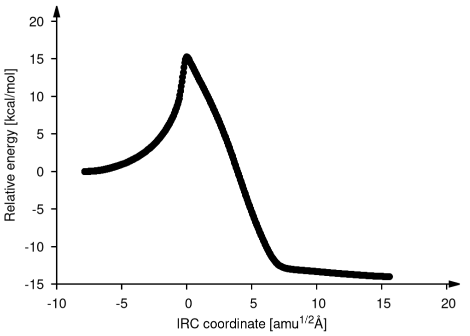

rxn38

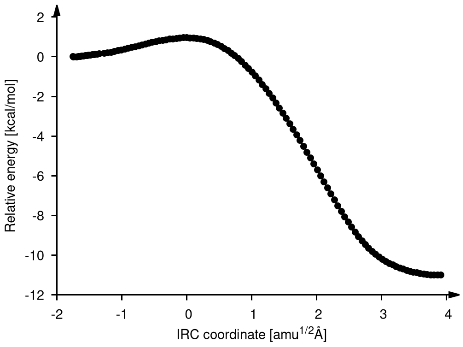

Figure S1: IRC plots from reaction 1 to 37 obtained using the PBE/DZVP-GGA/GEN-A2* level of theory. The M06 functional was used for reaction 38. The Zr atom of reaction 36 was treated with ECP. The uncorrected relative energy [kcal/mol] is plotted against the mass weighted IRC coordinate [amu$^{1/2}$Å].

S4. Comparison of the Local Coordinate and Gonzalez-Schlegel Methods for IRC calculations.

Figure S2 shows the differences between the intrinsic reaction coordinates (IRCs) for reactions 26 (left) and 32 (right) calculated with the local coordinate (LC) and Gonzalez-Schlegel (GS) methods. In reaction 26 LC and GS IRCs are quite similar except for the transition state (TS) region. Whereas the GS method connects the two IRC branches by a cusp a smooth TS region is found with the LC method. Closer inspection of the left graph in Figure S2 reveals further that the LC IRC energies are consistently lower than the GS one.

The right graph of Figure S2 compares the LC and GS IRCs for reaction 32. In this case the GS method fails completely for the description of the TS region. Furthermore, the IRC energy differences between the LC and GS method are now clearly visible.

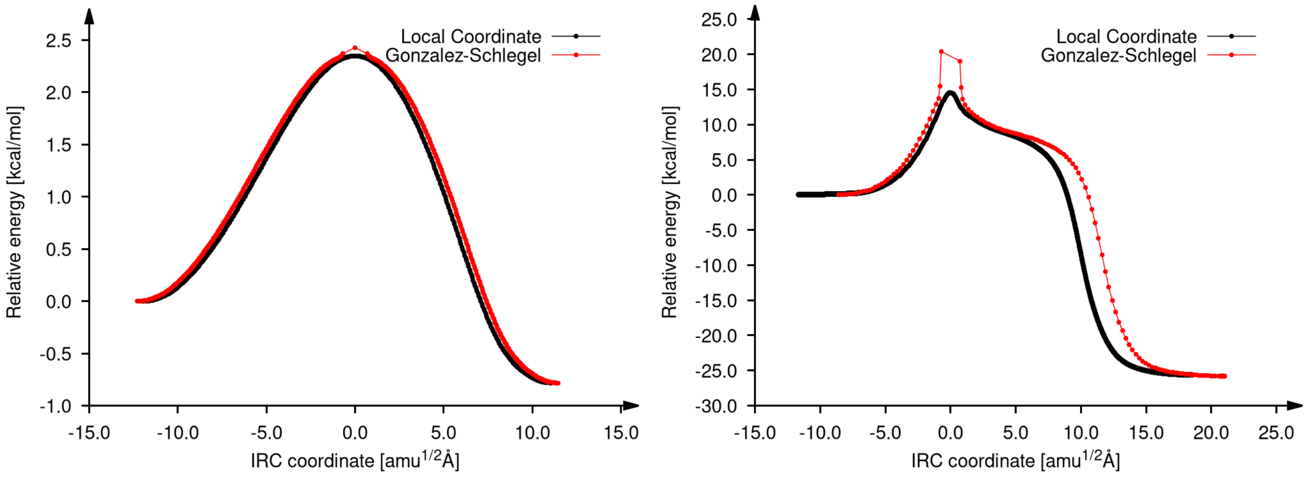

Figure S2: Comparison of the IRC plots for reaction 26 (left) and reaction 32 (right) using the local coordinate (LC) and Gonzalez-Schlegel (GS) methods. The uncorrected relative energy [kcal/mol] is plotted against the mass weighted IRC coordinate [amu$^{1/2}$Å].

Besides the TS regions the LC and GS methods can also show marked differences at the endpoints of the IRC. As example, we compare in Figure S3 the LC and GS IRC plots for reaction 4. For both IRC calculations the same convergence criteria (root mean square gradient equal to $1{\cdot}10^{-5}$ a.u.) were used. As Figure S3 shows the GS method converges at an earlier point as the LC one on the right side of the graph. As a result, the GS product structure is far from the corresponding local minimum whereas the corresponding LC structure is almost identical to it.

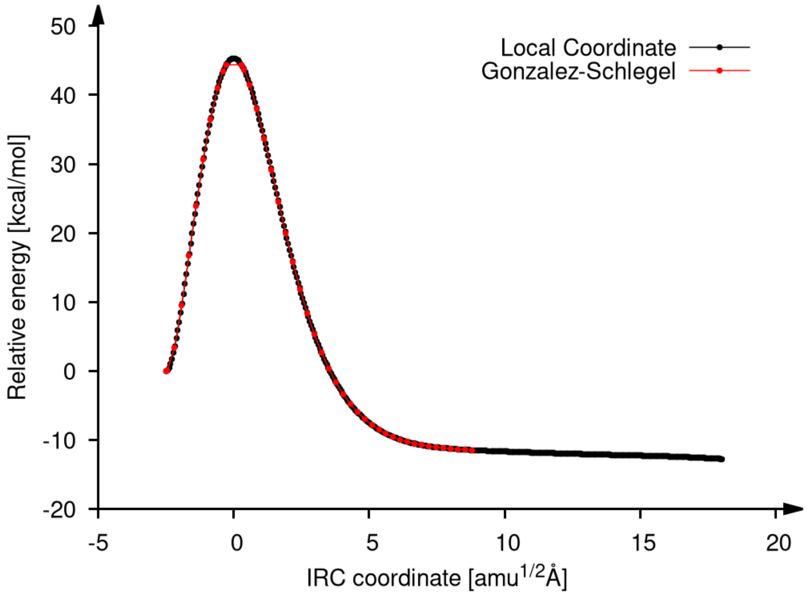

Figure S3: Comparison of the IRC plots for reaction 4 using the local coordinate (LC) and González-Schlegel (GS) methods. The uncorrected relative energy [kcal/mol] is plotted against the mass weighted IRC coordinate [amu$^{1/2}$Å].
